# Characterization of serum small extracellular vesicles and their small RNA contents across humans, rats, and mice

**DOI:** 10.1038/s41598-020-61098-9

**Published:** 2020-03-06

**Authors:** Fengbo Zhao, Li Cheng, Qian Shao, Zixing Chen, Xiufang Lv, Jing Li, Li He, Yufeng Sun, Qiuhong Ji, Peng Lu, Yuhua Ji, Juling Ji

**Affiliations:** 1 Department of Pathology, Medical School of Nantong University, Nantong, China; 2 Basic Medical Research Center, Medical School of Nantong University, Nantong, China; 3 Key Laboratory of Microenvironment and Translational Cancer Research, Nantong, China; 4 Key Laboratory of Neuroregeneration of Jiangsu and Ministry of Education, Nantong University, Nantong, China; 5 Institute of Immunology, College of Life Science and Technology, Jinan University, Guangdong, China; 6 Department of Neurology, Affiliated Hospital of Nantong University, Nantong, China

**Keywords:** Biomarkers, Medical research

## Abstract

Serum small extracellular vesicles (sEVs) have recently drawn considerable interest because of the diagnostic and therapeutic potential of their miRNAs content. However, the characteristics of human, mouse and rat serum sEVs and their differences in small RNA contents are still unknown. In this study, through nanoparticle tracking analysis and small RNA sequencing, we found that human, rat, and mouse serum sEVs exhibited distinct sizes and particle numbers as well as small RNA contents. Serum sEVs contained not only abundant miRNAs but also a large number of tRNA fragments. Most serum miRNAs existed both inside and outside of sEVs but were enriched in sEVs. Common serum sEV miRNAs (188 miRNAs) and species-specific serum sEV miRNAs (265, 58, and 159 miRNAs, respectively) were identified in humans, rats, or mice. The serum sEVs contained miRNAs from tissues and organs throughout the body, with blood cells as the main contributors. In conclusion, our findings confirmed the rationality of exploring serum sEV miRNAs as noninvasive diagnostic markers and revealed great differences in serum sEV small RNAs between humans, rats, and mice. Inadequate attention to these differences and the contribution of blood cells to serum sEV miRNAs could hinder the clinical translation of basic studies.

## Introduction

Circulating RNAs, especially microRNAs (miRNAs) have recently emerged as non-invasive disease biomarkers. miRNAs are endogenous small noncoding RNAs of approximately 22 nt that regulate gene expression posttranscriptionally by binding to target mRNA and repressing mRNA translation or increasing mRNA cleavage. Abnormally expressed miRNAs have been associated with multiple diseases. In 2008, Chen *et al*. reported that human serum contained numerous stable miRNAs that might originate in tissues throughout the body. The expression profiles of these miRNAs exhibit great potential to serve as novel noninvasive biomarkers for the diagnosis of cancer and other diseases^1^. To date, their study has been cited more than 4,000 times, which reflects the intensive interest of researchers in serum miRNAs as noninvasive biomarkers.

Exosomes are small extracellular vesicles (sEVs) (30–150 nm) that are secreted by fusion of multivesicular bodies to the plasma membrane^2^. These extracellular vesicles are functional vehicles carrying a complex cargo of proteins, lipids, and nucleic acids^3^. Serum exosomes are regarded as the main vector for circulating RNAs, and RNAs within exosomes are more stable and are protected from degradation by RNA enzymes^4^.

Increasing interest has been focused on serum exosome miRNAs as potential biomarkers for the detection of various cancers and other diseases. In the past decade, the number of publications on serum exosomal miRNAs has increased dramatically. However, there are still some questions that need to be addressed in the field. First, many studies addressing the diagnostic or therapeutic potential of serum exosomal miRNAs have been carried out with mice or rats models^5–7^, but to what extent the mouse and rat serum exosomal contents can resemble those of humans remains unclear. Additionally, prior to the prevalence of serum exosome studies, most studies related to blood miRNA markers were carried out in serum^1,8^, and the distribution of serum miRNAs inside and outside of exosomes is not well understood. Thus, how can we refer to these previous reports when we are studying serum exosomal miRNAs?

There is little available information regarding the differences between serum exosomes from humans, rats, and mice or between serum exosomes and exosome-free serum. Therefore, for the first time, the current study compared the small RNAs contents between different species as well as small RNAs contents inside and outside of serum exosomes and to provide clues for future studies on serum exosomal small RNAs.

## Results

### Identification and characterization of serum sEVs from humans, rats, and mice

**Figure 1:**
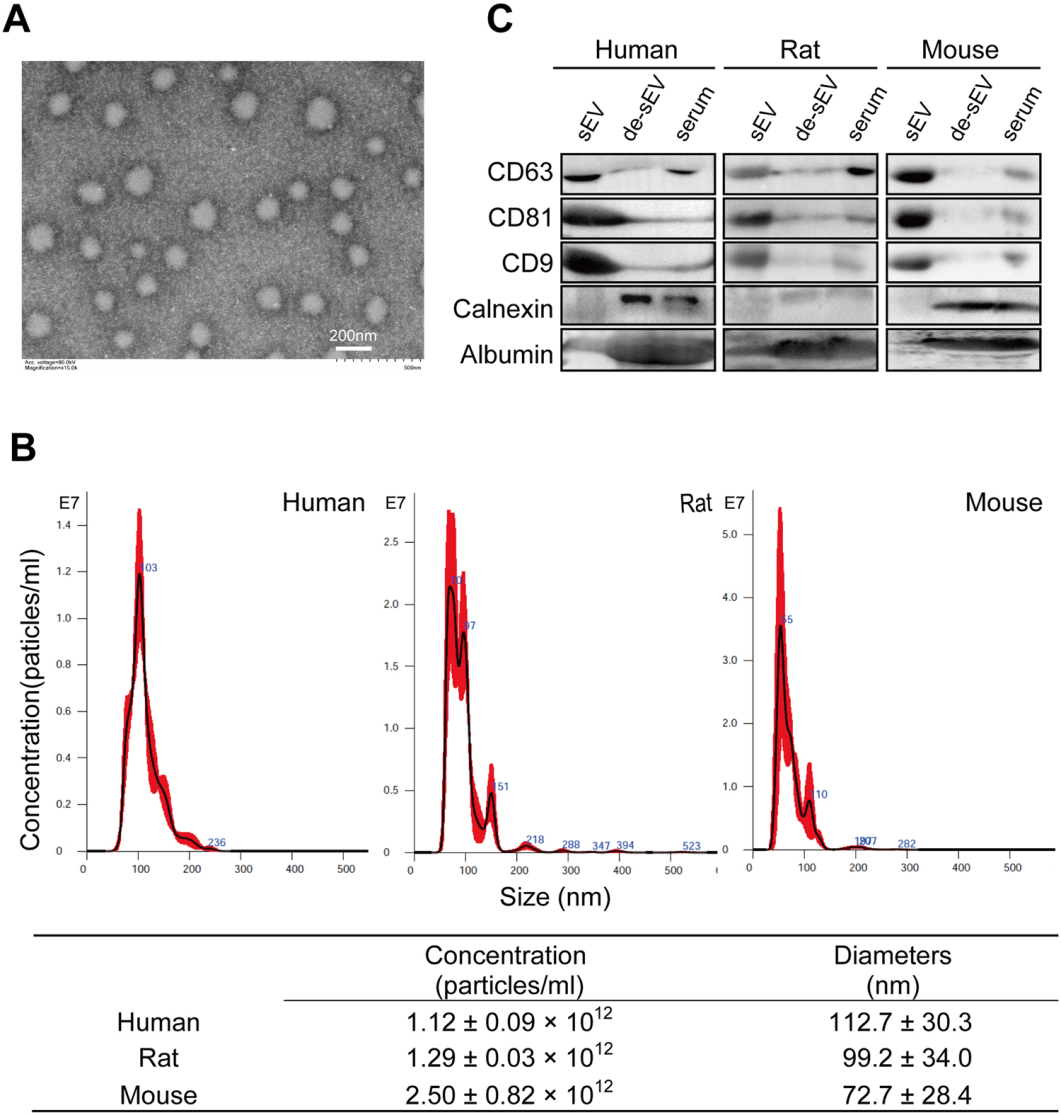
Characterization of sEV isolated from human, rat and mouse serum by Exoquick precipitation. (**A**) Transmission electron microscopy image of the isolated particles, bar = 200 nm, representative image of sEVs from rat. (**B**) Representative NTA size distribution profiles of isolated particles and their concentrations. (**C**) The expression of CD63, CD81 and CD9, as well as calnexin and albumin in the isolated particles was determined by western blotting. Lanes from left to right are serum sEV, de-sEV serum and whole serum, respectively. The images are the representatives of three or more independent experiments. Full-length blots/gels are presented in Supplementary Fig S2. sEV, small extracellular vesicle; de-sEV, small extracellular vesicle depleted.

Exosome-enriched extracellular vesicles (EVs) were precipitated from human, rat, and mouse serum. Under transmission electron microscopy (TEM), the isolated particles exhibited a round morphology and were uniform (Fig. 1A, see Supplementary Fig. S1 online). Nanoparticle tracking analyses (NTA) showed that the diameter of the exosome-enriched EVs ranged from 30–150 nm, which is consistent with the typical size of previously reported endocytic origin EVs (commonly known as exosomes) (Fig. 1B). According to MISEV 2018^9^, the obtained exosome-enriched EVs are referred to as “small EVs” (sEVs) hereafter based on their relatively small size.

In regarding to the three species, the size of human serum sEVs was the largest, followed by the sEVs isolated from rat serum, while those from mouse serum was the smallest (Fig. 1B), and compared with human and rat serum, more sEV particles were obtained from mouse serum (Fig. 1B). Notably, the number of particles in depleted serum was reduced to approximately 0.80%, 1.57%, and 0.35% of that in serum sEVs in humans, rats, and mice, respectively. Detailed information on the diameters and concentrations of serum sEVs and sEVs-depleted serum are provided in Supplementary Table S1.

To further confirm that the isolated particles were indeed exosome-enriched EVs, the expression of three tetraspanin proteins (CD63, CD81, and CD9) that are enriched in exosomes and are generally recommended as exosome biomarkers^9^, was examined by Western blotting. The bands of these markers were dark in isolated sEVs, but barely detectable in sEV-depleted serum (Fig. 1C, see Supplementary Fig. S2 online). To evaluate the potential contamination in the isolates, the expression of Calnexin, an endoplasmic reticulum marker, and Albumin, the highest abundant proteins in the serum, were also detected by Western blotting. Compared with the dark bands of these two proteins in serum and sEVs-depleted serum, only faint bands could be observed in sEV fractions in all the three species, indicating that there was little contamination of vesicles from the endoplasmic reticulum and serum proteins in the isolated particles.

In short, the above observations proved that the methods for serum sEV isolations in the present study was effective and reliable, and suggested the size and concentration of serum sEVs differed among the three species.

### Comparison of small RNA contents between human, rat and mouse serum sEVs

**Figure 2:**
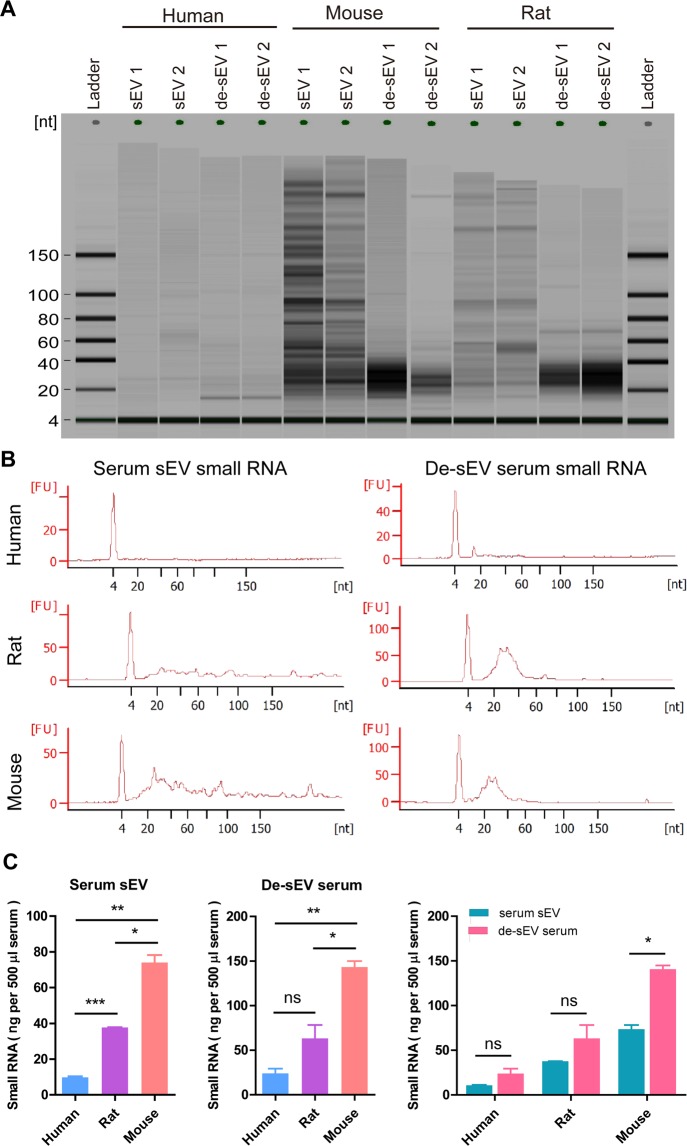
The length distribution and concentration of small RNAs in serum sEV and de-sEV serum samples from human, rats and mice as detected by Agilent 2100 Bioanalyzer using small RNA chips. (**A**) Gel-like image of all the samples with biological replicates. (**B**) For each group, electropherograms for one of the two biological replicates was provided. (**C**) The concentrations of small RNA (0–296nt) in serum sEV from human, rat and mouse, human vs. rat, ***P = 0.0003; human vs. mouse, **P = 0.0053; rat vs. mouse, *P = 0.0159; de-sEV serum, human vs. rat, P > 0.05, ns; human vs. mouse, **P = 0.0052; rat vs. mouse, *P = 0.0397; the concentrations of small RNA in serum sEV vs. de-sEV serum, human, P > 0.05, ns; rat, P > 0.05, ns; mouse, *P = 0.0134. sEV, small extracellular vesicle; de-sEV, small extracellular vesicle depleted.

Circulating small RNAs were not restricted to vesicles. The concentration and size distribution of the small RNAs in serum sEVs and sEV-depleted serum were determined with an Agilent Bioanalyzer 2100 (Fig. 2A,B). We found several differences among the three species. The first and the most significant difference was the small RNA concentration in both serum sEVs and sEV-depleted serum was much lower in humans, and the difference between humans and mice was significant (Fig. 2C). Second, the size of RNAs in rat and mouse serum sEVs distributed in a relatively wide range (20–150 nt), while those in sEV-depleted serum were mainly limited to the range of small RNA (20 to 40 nt) (Fig. 2B). Finally, although the pretreatment and precipitation kits effectively removed serum EVs (see Supplementary Table S1 online), sEV-depleted serum contained even more small RNAs than serum sEVs in all three species, and the difference was significant in mice (Fig. 2C). These RNAs might be combined with lipoproteins^10^ or protein complexes^11^ and are protected from RNase.

**Figure 3:**
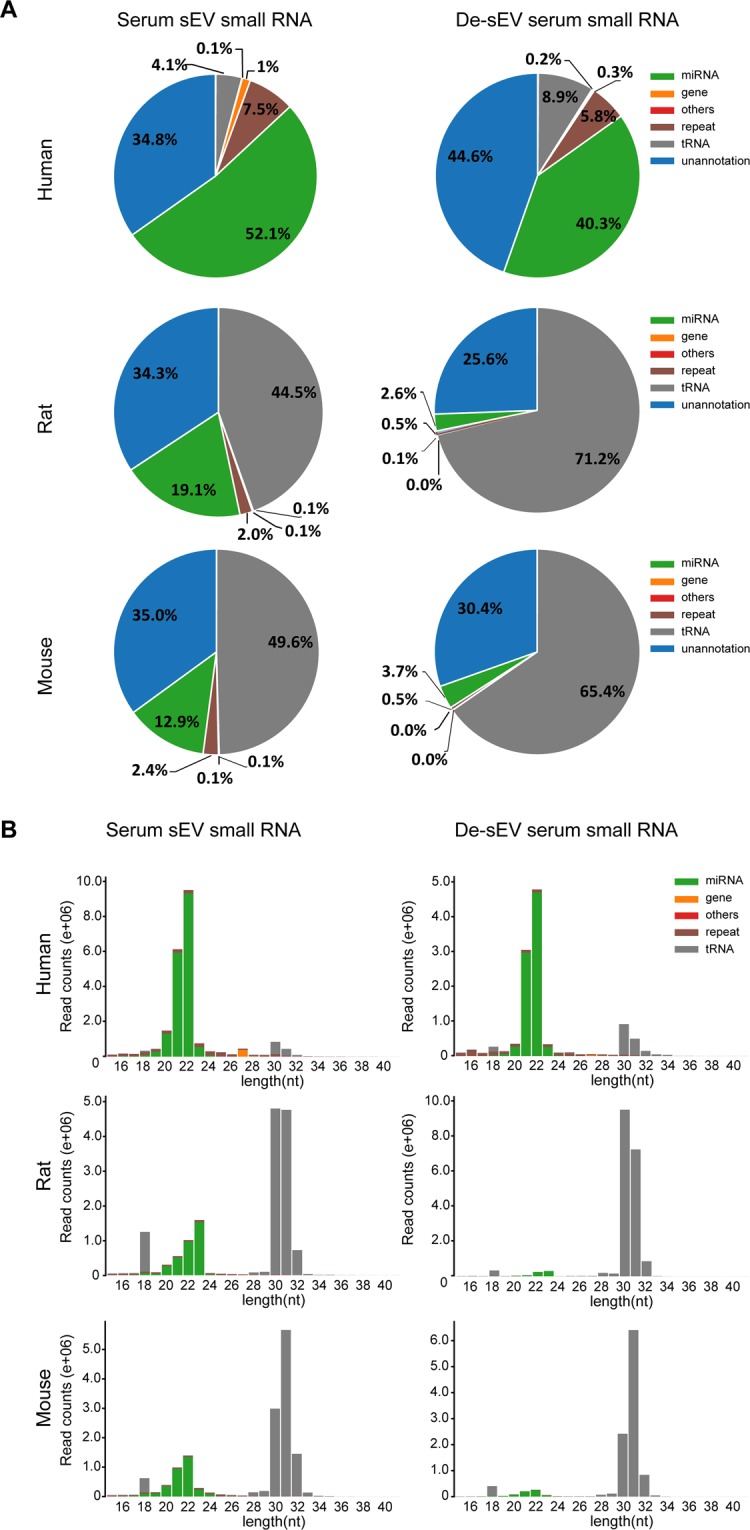
The length distribution and frequency of annotated reads of small RNAs in serum sEV samples and de-sEV serum samples from human, rats and mice. (**A**) Length distribution of the clean reads. (**B**) Pie charts of annotated small RNA species and their percentages in total clean reads, others including: rRNA, snRNA, Cis-reg and other_Rfam_RNA. For each group, one representative chart of the two replicates was provided. Detailed annotation data for all the samples were provided in Supplementary Table S3. sEV, small extracellular vesicle; de-sEV, small extracellular vesicle depleted.

Then, small RNA sequencing was performed to elaborate the differences in the RNA species of serum sEVs and sEV-depleted serum in humans, rats and mice. According to the annotated data, miRNAs and tRNA (tRFs & tiRNAs) were the two main types of small RNA species in serum sEVs across species (Fig. 3A). tRFs (tRNA-derived fragments) and tiRNAs (tRNA halves) are tRNA-derived noncoding RNAs. Very low proportions of ribosomal RNA (rRNA), small nuclear RNA (snRNA), and cis-reg RNA were also detected. Detailed sequencing and annotation data are provided in Supplementary Table S2 and Supplementary Table S3.

The length of these annotated small RNAs from both serum sEVs and sEV-depleted serum formed two peaks at approximately 20–24 nt and 30–32 nt. After overlying the reads of the identified small RNAs on their length distributions, we could find miRNAs were predominantly distributed between 20–24 nt, while tRNA (tRFs & tiRNAs) constituted the other peak at 30–32 nt (Fig. 3B).

Again, the proportion of small RNA components varied among species. Interestingly, although the amount of small RNA in human sEVs and sEV-depleted serum was the lowest (Fig. 2C), their proportion of miRNAs was the highest among the three species (Fig. 3). In contrast, tRNAs (tRFs & tiRNAs) were the overwhelmingly dominant small RNAs in both sEVs and sEV-depleted serum from mice and rats (Fig. 3).

### The miRNA profiles of serum sEVs and sEV-depleted serum in human, rat and mouse

**Figure 4:**
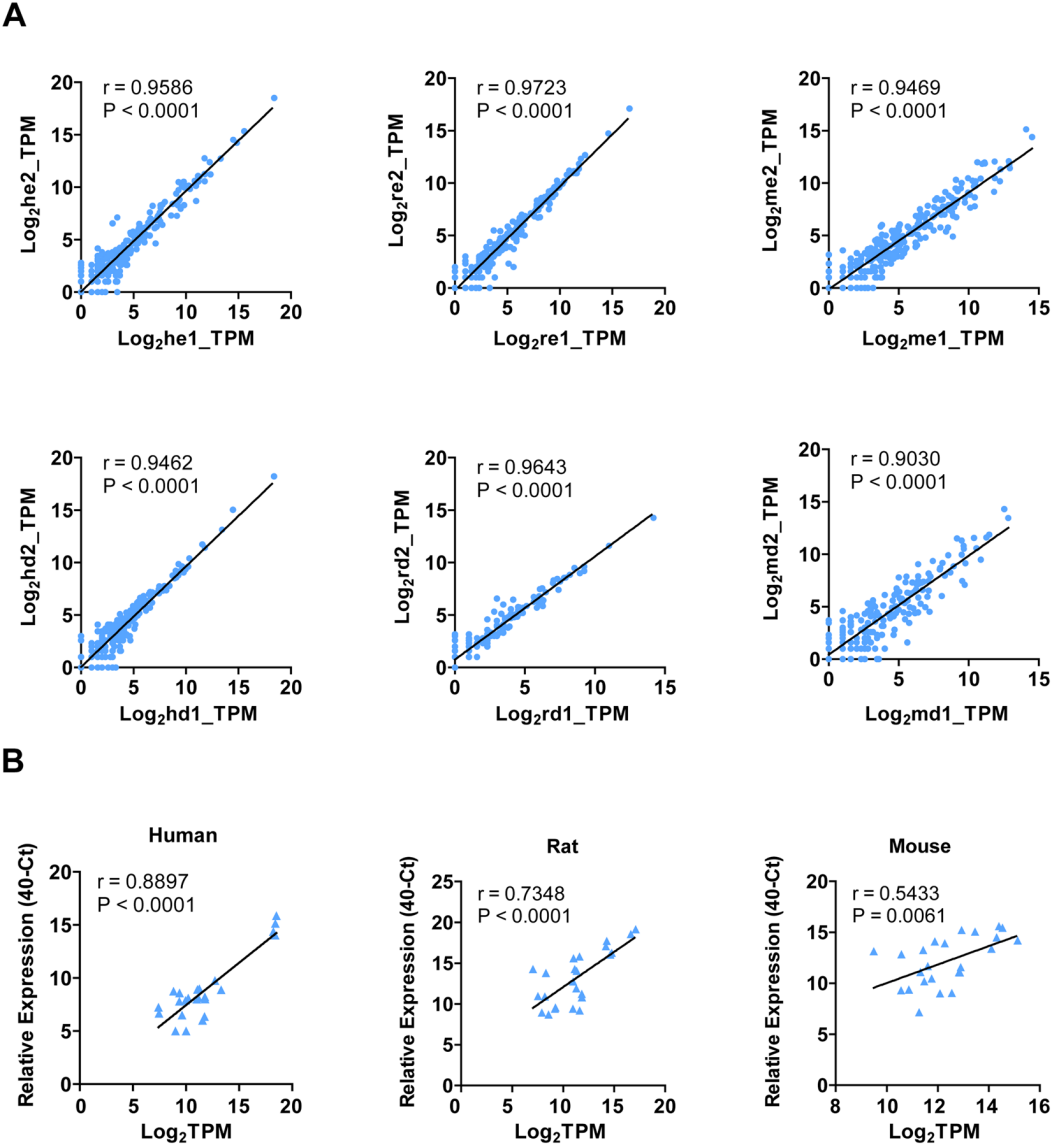
Profiling of human, rat, and mouse serum sEV miRNAs. (**A**) Correlation between normalized miRNAs read counts (Log_2_TPM) between the biological replications from each group. (**B**) Correlation between normalized read counts (Log_2_TPM) obtained by small RNA-seq and adjusted q-RT-PCR CT values for 6 miRNAs (miR-125a-5p, miR-125b-5p, miR-191-5p, miR-27b-3p, miR-486-5p and miR-99a-5p). q-RT-PCR for miRNAs were performed with the same batch of RNAs that prepared for sequencing. The relative expression of the selected miRNAs as determined by qPCR were expressed as (40-Ct) miRNA. TPM, transcripts per million reads; h, human; r, rat; m, mouse; e, serum small extracellular vesicle; d, small extracellular vesicle depleted serum.

Circulating miRNAs are the focus of numerous biomarker discovery studies. We then shift our attention to the miRNAs profile of sEVs and sEV-depleted serum. Because the abundance of most miRNAs was low, we defined detectable miRNAs as those that had at least one transcript per million reads (TPM ≥ 1). Accordingly, a total of 500, 319, and 446 known miRNAs were identified from human, rat, and mouse serum sEVs, respectively, and a total of 331, 189, and 306 known miRNAs were identified from sEV-depleted serum (see Supplementary Table S4 online). The biological replications were highly correlated in each group (Fig. 4A). The sequencing data were further validated by qRT-PCR analysis of 6 miRNAs (miR-125a-5p, miR-125b-5p, miR-191-5p, miR-27b-3p, miR-486-5p and miR-99a-5p) with differential expression levels. As showed in Fig. 4B, the Log2 transformed TPM values of the six miRNAs were highly correlated with their corresponding relative expression levels of PCR analysis in all the three species.

### miRNAs inside and outside of serum sEVs

**Figure 5:**
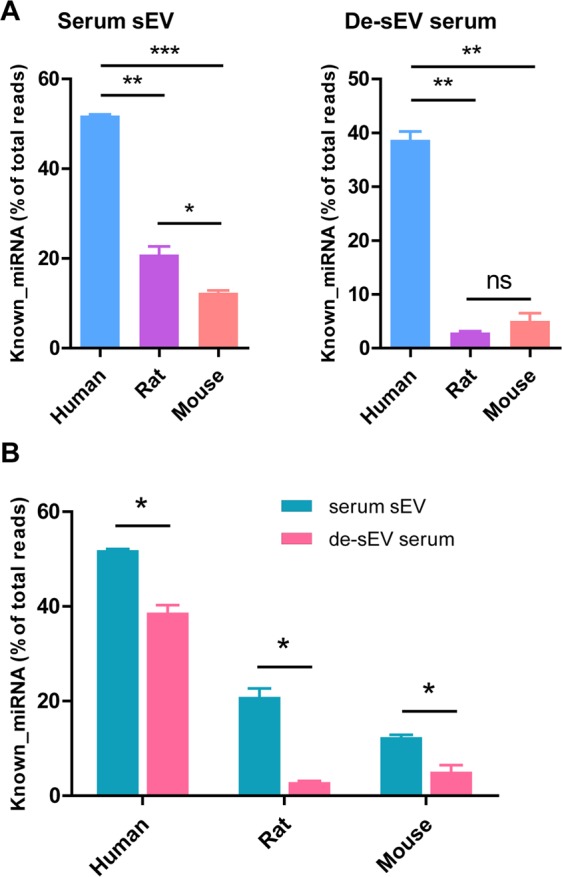
The frequency of known miRNAs among total clean reads of small RNAs. (**A**) The frequency of known miRNAs in serum sEVs from human, rat and mouse, human vs. rat, **P = 0.0035; human vs. mouse, ***P = 0.0002; rat vs. mouse, *P = 0.0458; de-sEV serum, human vs. rat, **P = 0.0019; human vs. mouse, **P = 0.0039; rat vs. mouse, ns, P = 0.2693. (**B**) The frequency of known miRNAs in serum sEVs vs. de-sEV serum, human, *P = 0.014; rat, *P = 0.0103; mouse, *P = 0.0399. sEV, small extracellular vesicle; de-sEV, small extracellular vesicle depleted.

It is well acknowledged that sEVs are the primary form of circulating miRNAs; however, our data suggested that a considerable proportion of miRNAs existed in sEV-depleted serum (Fig. 5). Moreover, the proportion of known miRNAs in human serum sEVs was higher than those in rats and mice (human vs. rat, P < 0.01; human vs. mouse, P < 0.001), and it was also higher in rats than in mice (P < 0.05). In sEV-depleted serum, the proportion of known miRNAs in humans was still higher than those in rats and mice (P < 0.01), but no difference was found between rats and mice (Fig. 5A).

We further compared the frequency of known miRNAs between serum sEVs and sEV-depleted serum, and it was found that miRNAs were enriched in serum sEVs (P < 0.05), especially in rats and mice. The frequencies of known miRNAs in serum sEVs were approximately 7.22 and 2.44 times those in sEV-depleted serum in rats and mice, respectively, versus 1.34 times in humans (Fig. 5B).

**Figure 6:**
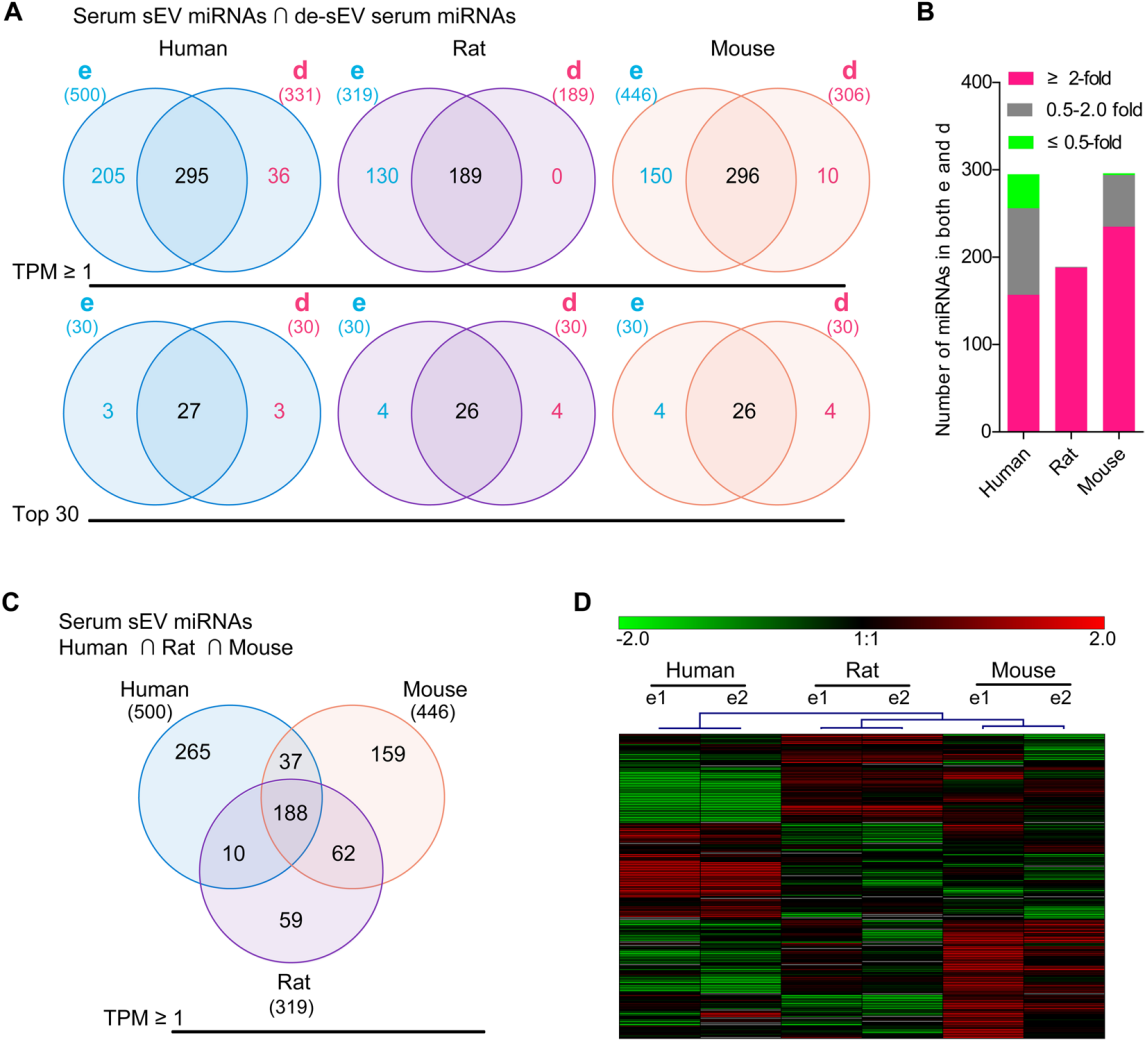
The expression of miRNAs in serum sEV vs. de-sEV serum, and the expression of miRNAs in serum sEV between species. (**A**) The venny diagram depicts the common and unique miRNAs identified in serum sEV miRNAs and de-sEV serum miRNAs from human, rat and mouse, respectively. (**B**) The composition of miRNAs based on the relative expression of serum sEVs vs. sEV-depleted serum, only those miRNAs existed both inside and outside of serum sEV were included. (**C**) The venny diagram depicts the common and unique miRNAs identified in serum sEV miRNAs from human, rat and mouse. (**D**) Heat map of hierarchical clustering of the 188 common serum sEV miRNAs between species based on log2 transformed TMP (transcripts per million reads). Each column represents an individual sample, each row represents an individual miRNA. miRNAs were ordered by Pearson correlation and complete linkage. TPM, transcripts per million reads; sEV, small extracellular vesicle; de-sEV, small extracellular vesicle depleted; e, serum small extracellular vesicle; d, small extracellular vesicle depleted serum.

We then asked if there were any miRNAs that existed exclusively inside or outside of serum sEVs. After comparing the miRNA profiles of serum sEVs and sEV-depleted serum from humans, rats and mice, we found that there were more known miRNA species in sEVs than sEVs-depleted serum in all three species, and most of the miRNAs identified in sEVs depleted serum also existed inside sEVs: human, 89.1%; rat, 100%; mouse, 96.7% (Fig. 6A, see Supplementary Table S5 online). However, the miRNAs that existed exclusively inside or outside of sEVs were generally of low abundance (see Supplementary Table S5 online). Only four miRNAs that were exclusively detected in human serum sEVs (miR-450b-5p, miR-374a-5p, miR-942-5p, miR-576-5, TPM ranged from 12.5 to 15.0) and one (mmu-miR-98-5p, TPM = 27.5) in mouse sEVs exhibited a TPM ≥ 10; these miRNAs could be recognized as sEV-specific miRNAs, and none of the miRNAs that appeared exclusively outside of sEVs exhibited a TPM ≥ 10.

We also noted that among those miRNAs that presented both inside and outside of serum sEVs, miRNA expression was enriched inside serum sEVs, especially in rats and mice (Fig. 6B). In humans, among the 295 miRNAs, the TPM values of 157 miRNAs (53.2%) in serum sEVs were higher than those in sEV-depleted serum (≥2-fold), while only 39 miRNAs (13.2%) showed lower TPM values inside serum sEVs (≤0.5-fold). In rats, among the 189 miRNAs, the TPM values of 188 miRNAs (99.5%) in serum sEVs were higher than those in sEV-depleted serum (≥3-fold), and only one miRNA showed equal expression in the two sample types; in mice, among the 296 miRNAs, the TPM values of 235 miRNAs (79.4%) in serum sEVs were higher than those in sEV-depleted serum (≥2-fold), and only two miRNAs (0.67%) showed lower TPM values inside serum sEVs (0.5-fold). The expression data for the top 30 serum sEV miRNAs are provided in Table 1.

**Table 1:** Expression of the top 30 serum sEV miRNAs in humans, rats, and mice vs. corresponding de-sEV serum (Top 30 for each species).

Human				Rat				Mouse			
miRNA_id	he_TPM	hd_TPM	Fold-change	miRNA_id	re_TPM	rd_TPM	Fold-change	miRNA_id	me_TPM	md_TPM	Fold-change
hsa-miR-486-5p	360870.0	325558.5	1.1	rno-miR-191a-5p	121844.0	19317.0	6.3	mmu-miR-191-5p	26739.5	13145.5	2.0
hsa-miR-92a-3p	44958.0	10094.5	4.5	rno-miR-486	26388.0	2597.0	10.2	mmu-miR-486a-5pmmu-miR-486b-5p	22677.0	9291.0	2.4
hsa-miR-451a	24460.0	3169.0	7.7	rno-miR-423-5p	6009.5	699.0	8.6	mmu-miR-99a-5p	5905.0	2797.5	2.1
hsa-miR-423-5p	23392.0	28184.0	0.8	rno-miR-26a-5p	4620.0	548.5	8.4	mmu-miR-125a-5p	5563.0	3295.0	1.7
hsa-miR-191-5p	8452.0	3218.0	2.6	rno-miR-125a-5p	3643.5	596.5	6.1	mmu-miR-27b-3p	5312.0	1295.0	4.1
hsa-let-7b-5p	5221.5	1320.5	4.0	rno-miR-22-3p	3148.0	378.0	8.3	mmu-miR-10b-5p	3986.5	2184.5	1.8
hsa-miR-26a-5p	5138.0	633.0	8.1	rno-miR-10a-5p	2675.0	300.5	8.9	mmu-miR-125b-5p	3762.0	1518.0	2.5
hsa-miR-10b-5p	3795.5	703.5	5.4	rno-miR-99a-5p	2572.0	311.5	8.3	mmu-miR-146a-5p	3132.0	1145.0	2.7
hsa-miR-10a-5p	2990.0	465.5	6.4	rno-miR-146a-5p	2488.0	255.0	9.8	mmu-miR-22-3p	3033.5	859.0	3.5
hsa-miR-125b-5p	2983.5	986.0	3.0	rno-miR-27b-3p	2416.5	225.0	10.7	mmu-miR-26a-5p	2978.0	461.0	6.5
hsa-miR-99a-5p	2537.0	799.0	3.2	rno-miR-128-3p	2305.0	197.0	11.7	mmu-miR-10a-5p	2845.5	1629.5	1.7
hsa-miR-146a-5p	2269.5	425.5	5.3	rno-miR-125b-5p	2252.0	240.5	9.4	mmu-miR-423-5p	2831.5	1744.0	1.6
hsa-miR-125a-5p	2213.5	830.0	2.7	rno-miR-215	2124.0	499.5	4.3	mmu-miR-148a-3p	2587.0	720.5	3.6
hsa-miR-22-3p	1927.0	610.0	3.2	rno-miR-143-3p	1700.5	132.5	12.8	mmu-miR-143-3p	2090.0	488.5	4.3
hsa-miR-30d-5p	1903.0	530.5	3.6	rno-miR-23a-3p	1281.5	122.0	10.5	mmu-miR-99b-5p	1616.5	667.5	2.4
hsa-miR-25-3p	1686.5	560.0	3.0	rno-miR-99b-5p	1111.5	117.5	9.5	mmu-miR-30d-5p	1297.5	1294.0	1.0
hsa-miR-100-5p	1560.5	472.5	3.3	rno-miR-146b-5p	1028.0	78.0	13.2	mmu-miR-25-3p	1222.5	429.5	2.8
hsa-miR-122-5p	1213.0	778.0	1.6	rno-let-7f-5p	929.0	51.5	18.0	mmu-miR-23a-3p	1204.0	331.5	3.6
hsa-miR-99b-5p	1187.0	429.5	2.8	rno-let-7g-5p	908.5	53.5	17.0	mmu-miR-100-5p	1149.5	478.0	2.4
hsa-let-7a-5p	1178.0	188.0	6.3	rno-miR-25-3p	873.0	88.0	9.9	mmu-miR-192-5p	1079.5	362.5	3.0
hsa-miR-484	1148.0	794.0	1.4	rno-miR-192-5p	798.5	169.5	4.7	mmu-miR-128-3p	906.0	309.0	2.9
hsa-miR-363-3p	1128.5	282.0	4.0	rno-miR-92a-3p	785.0	84.0	9.3	mmu-let-7g-5p	886.5	46.5	19.1
hsa-miR-320a	1029.5	451.0	2.3	rno-let-7d-3p	745.0	76.0	9.8	mmu-miR-21a-5p	878.0	214.5	4.1
hsa-let-7f-5p	970.5	153.5	6.3	rno-let-7a-5p	640.5	37.5	17.1	mmu-let-7c-5p	800.0	67.0	11.9
hsa-miR-151a-5p	962.5	222.5	4.3	rno-miR-100-5p	629.0	73.0	8.6	mmu-let-7i-5p	782.0	43.5	18.0
hsa-let-7d-3p	738.0	91.0	8.1	rno-miR-320-3p	623.5	81.5	7.7	mmu-miR-451a	769.5	283.5	2.7
hsa-miR-96-5p	608.5	121.5	5.0	rno-miR-151-3p	616.5	74.5	8.3	mmu-miR-151-3p	704.0	414.5	1.7

**Note:** h, human; r, rat; m, mouse; sEV, small extracellular vesicle; de-sEV, small extracellular vesicle depleted; e, serum small extracellular vesicle; d, small extracellular vesicle-depleted serum; TPM, transcripts per million reads. The TPM values provided here are the average TPM values of two biological replicates.

### The common and species-specific serum sEV miRNAs among human, rat and mouse

To determine the common and species-specific serum sEV miRNAs, the human, rat, and mouse sEV miRNA profiles were aligned according to miRNA IDs and the corresponding sequences. A total of 188 common miRNAs were identified in all three species (Fig. 6C, see Supplementary Table S6-1 online). Although these 188 miRNAs were common across the species, their expression patterns were distinct (Fig. 6D). The top 30 common miRNAs based on the abundance of human serum sEV miRNAs are provided in Table 2. Accordingly, 265, 59, and 159 miRNAs that were detectable exclusively in humans, rats, or mice were defined as species-specific serum sEV miRNAs (Fig. 6C, see Supplementary Table S6-2 online).

**Table 2:** The top 30 common miRNAs in human, rat and mouse serum sEVs (based on the TPM values of human serum miRNAs).

miRNA_id	Human	Rat	Mouse
	he_TPM	re_TPM	me_TPM
miR-486-5p	360870.0	26388.0	22677.0
miR-92a-3p	44958.0	785.0	445.5
miR-451a	24460.0	391.5	769.5
miR-423-5p	23392.0	6009.5	2831.5
miR-191-5p	8452.0	121844.0	26739.5
let-7b-5p	5221.5	524.0	568.5
miR-26a-5p	5138.0	4620.0	2978.0
miR-10b-5p	3795.5	9.0	3986.5
miR-10a-5p	2990.0	2675.0	2845.5
miR-125b-5p	2983.5	2252.0	3762.0
miR-99a-5p	2537.0	2572.0	5905.0
miR-146a-5p	2269.5	2488.0	3132.0
miR-125a-5p	2213.5	3643.5	5563.0
miR-22-3p	1927.0	3148.0	3033.5
miR-30d-5p	1903.0	526.5	1297.5
miR-25-3p	1686.5	873.0	1222.5
miR-100-5p	1560.5	629.0	1149.5
miR-122-5p	1213.0	236.0	79.5
miR-99b-5p	1187.0	1111.5	1616.5
let-7a-5p	1178.0	640.5	455.5
miR-484	1148.0	192.5	628.5
miR-363-3p	1128.5	79.5	24.5
miR-320a	1029.5	623.5	418.0
let-7f-5p	970.5	929.0	618.0
miR-151a-5p	962.5	180.0	293.0
let-7d-3p	738.0	745.0	596.5
miR-96-5p	608.5	8.5	25.5
miR-423-3p	607.5	489.5	417.0
miR-27b-3p	562.5	2416.5	5312.0
miR-148a-3p	527.0	204.5	2587.0

Note: h, human; r, rat; m, mouse; sEV, small extracellular vesicle; e, serum small extracellular vesicle; TPM, transcripts per million reads. The TPM values provided here are the average of two biological replicates. Details are provided in Supplementary Table S6-1.

Common serum miRNAs were generally highly expressed; 108 out of 188 miRNAs in humans (57.4%), 115 out of 188 miRNAs in rats (61.2%), and 125 out of 188 miRNAs in mice (66.5%) presented a TPM ≥ 10. However, most of the species-specific miRNAs were low in abundance. Only 37 out of 265 human-specific miRNAs (14.5%), 9 out of 59 rat-specific miRNAs (15.5%), and 13 out of 159 mouse-specific miRNAs exhibited a TPM ≥ 10 (8.2%). The species-specific serum sEV miRNAs with relatively high abundance (TPM ≥ 10) are provided in Table 3.

**Table 3:** Human-, rat- or mouse-specific serum sEV miRNAs that were exclusively detected in each species (TPM ≥ 10).

Human				Rat		Mouse	
miRNA_id	he_TPM	miRNA_id	he_TPM	miRNA_id	re_TPM	miRNA_id	me_TPM
miR-197-3p	249.0	miR-664a-5p	28.0	miR-215	2124.0	miR-145a-3p	295.5
miR-584-5p	188.0	miR-323b-3p	27.5	miR-191a-3p	224.0	miR-1198-5p	137.5
miR-4732-3p	134.0	miR-629-5p	25.0	miR-3557-5p	103.5	miR-676-3p	76.0
miR-151a-3p	124.0	miR-1908-5p	16.0	miR-182	65.5	miR-1964-3p	24.0
miR-486-3p	105.5	miR-374a-5p	15.0	miR-676	20.5	miR-28a-3p	22.0
miR-3615	99.0	miR-3605-3p	14.5	miR-205	20.0	miR-486a-3p	18.5
miR-885-5p	81.5	miR-424-3p	14.0	miR-191b	16.0	miR-1943-5p	18.0
miR-2110	79.5	miR-320d	13.5	miR-374-5p	14.5	miR-378c	15.5
miR-500a-3p	62.0	miR-4685-3p	13.5	miR-488-3p	12.0	miR-1983	15.0
miR-4732-5p	60.5	miR-1226-3p	13.5			miR-429-3p	15.0
miR-502-3p	56.0	miR-1285-3p	13.0			miR-669c-5p	11.5
miR-320c	51.0	miR-942-5p	13.0			miR-1981-5p	11.0
miR-1180-3p	49.0	miR-625-3p	13.0			miR-21a-3p	10.0
miR-320b	46.5	miR-576-5p	12.5				
miR-654-3p	43.5	miR-769-5p	12.5				
miR-941	42.5	miR-550a-5p	12.0				
miR-589-5p	39.5	miR-3184-5p	11.5				
miR-4433b-5p	37.5	miR-432-5p	11.0				
miR-1301-3p	36.5						

Note: h, human; r, rat; m, mouse; sEV, small extracellular vesicle; e, serum small extracellular vesicle; TPM, transcripts per million reads. The TPM values provided here are the average of two biological replicates. Details are provided in Supplementary Table S6-2.

### Top ranked serum sEV miRNAs and their plausible sources

**Figure 7:**
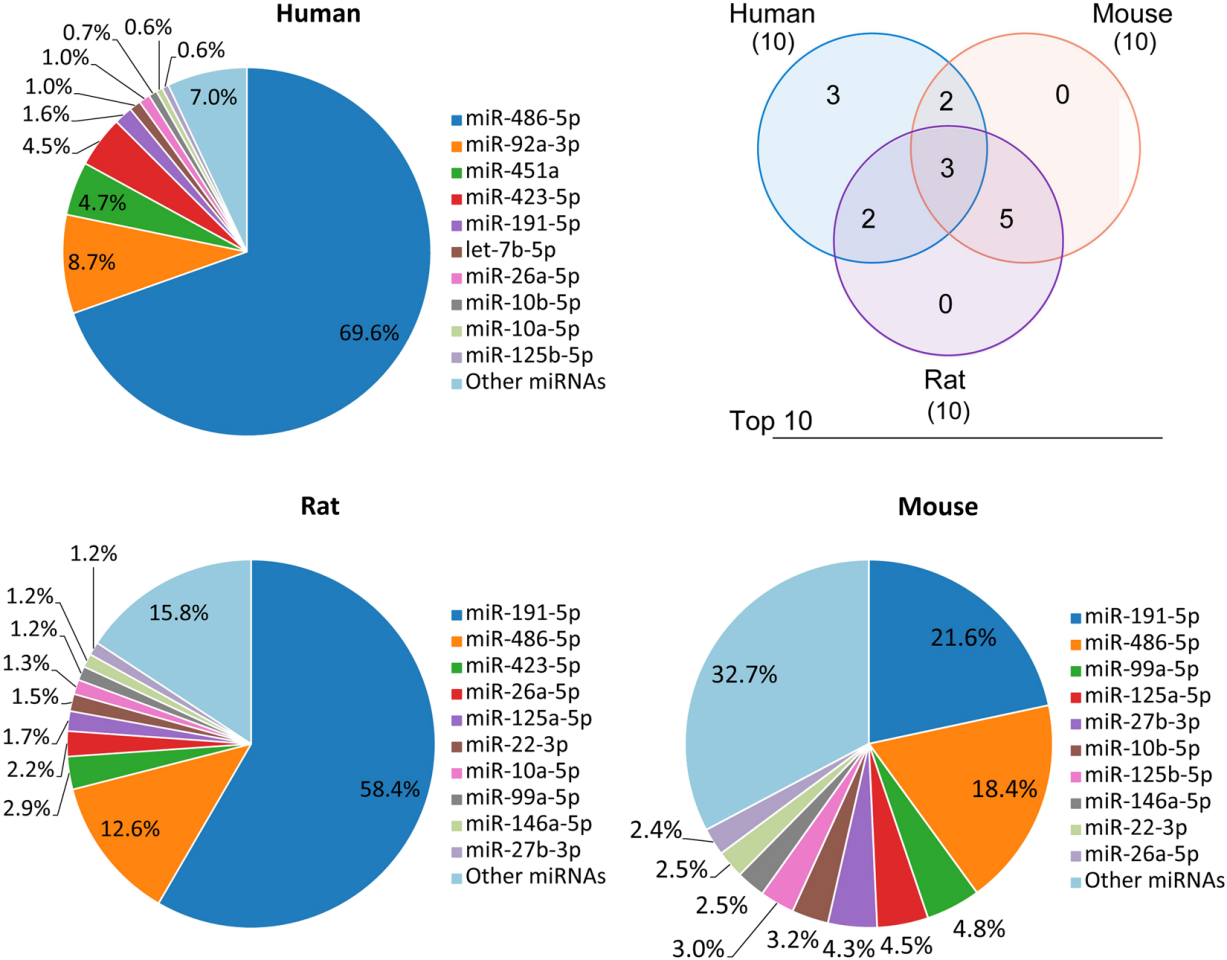
Pie charts of the top ten most abundant serum sEV miRNAs in human, rat and mice, and venny diagram depicts the common and unique miRNAs identified in the top ten most abundant serum sEV miRNAs from human, rat and mouse.

The most abundant serum sEV miRNA in humans was miR-486-5p, while that in rats and mice was miR-191-5p. Surprisingly, these miRNAs accounted for 69.6%, 58.4%, and 21.6% of all detectable miRNAs in humans, rats and mice, respectively. The top 10 most abundant miRNAs accounted for 93.0%, 84.2% and 67.3% of all detectable miRNAs in humans, rats, and mice, respectively (Fig. 7). Among these top 10 miRNAs, humans shared 5 with either rats or mice, while rats and mice shared 8 with each other.

To analyze the possible sources of serum sEV miRNAs, the cell types and tissue enrichment of the top 10 human serum sEV miRNAs were annotated according to the DASHR v2.0: database (http://dashr2.lisanwanglab.org/)^12^ (Table 4). miR-486-5p, the top human serum sEV miRNA, was exclusively detected in erythrocytes; miR-92a-3p, the second-ranked miRNA, was highly expressed in mature erythrocytes, CD4 + T cells, monocyte-derived macrophages and the brain and lungs; miR-451a, the third-ranked miRNA, was highly expressed in peripheral blood mononuclear cells and lung, adipose, skin, and breast tissues. Thus, blood cells, including mature erythrocytes, CD4 + T cells and monocyte-derived macrophages, were the main contributors to the serum sEV miRNAs. Some serum sEV miRNAs come from certain tissues and organs throughout the body, including adipose, skin, brain, lung, breast, pancreatic islet, colon, kidney, prostate, ventricular-myocardium, fibroblast and gastric tissues.

**Table 4:** The cell types and tissue enrichment of the top 10 human serum sEV miRNAs.

miRNA_id	he_TPM	hd_TPM	Fold-change	Cell or Tissue enriched
hsa-miR-486-5p	360870.0	325558.5	1.1	**mature-erythrocyte**
hsa-miR-92a-3p	44958.0	10094.5	4.5	**mature-erythrocyte**, brain pfc, lung, **CD4 + Tcell, monocyte-derived-macrophage**
hsa-miR-451a	24460.0	3169.0	7.7	lung, adipose, skin, breast, **peripheral bmc**
hsa-miR-423-5p	23392.0	28184.0	0.8	testicular germ, pancreatic beta cell, pancreatic islet
hsa-miR-191-5p	8452.0	3218.0	2.6	brain pfc, brain sfg, **monocyte-derived-macrophage**
hsa-let-7b-5p	5221.5	1320.5	4.0	testicular germ, skin, colon, kidney
hsa-miR-26a-5p	5138.0	633.0	8.1	brain sfg
hsa-miR-10b-5p	3795.5	703.5	5.4	adipose, skin, kidney, colon
hsa-miR-10a-5p	2990.0	465.5	6.4	lung, colon, skin, adipose
hsa-miR-125b-5p	2983.5	986.0	3.0	prostate, ventricular-myocardium, lung, primary fibroblast, gastric

Note: brain pfc, brain prefrontal cortex; brain sfg, brain superior frontal gyrus; peripheral bmc, peripheral blood mononuclear cells.; h, human; sEV, small extracellular vesicle; e, serum small extracellular vesicle; d, small extracellular vesicle-depleted serum; TPM, transcripts per million reads. The TPM values provided here are the average of two biological replicates.

We also sought to explore the tissue-specific distribution of serum sEV miRNAs in rats and mice. In mice, the available data on the tissue-specific distribution of miRNAs are very limited. For rats, there is an available miRNA expression atlas from male rats^13^, but it only includes miRNA expression data from the parenchyma organ. Among the top 2 miRNAs, miR-191-5p was only detectable in the rat lymph nodes, spleen, and thymus, while miR-486-5p was not detected in any of the parenchymal organs. The other miRNAs showed a variety of tissue origins (see Supplementary Table S7 online).

## Discussion

In this study, serum sEVs from humans, rats and mice were isolated and characterized. The small RNA contents as well as the miRNA contents of serum sEVs and in sEV-depleted serum were compared among healthy humans, rats and mice by small RNAseq analysis.

High recovery and reproducibility of sEV isolation are very important for the translational study of serum sEVs; therefore, the data obtained by different research groups may be comparable. Based on our previous studies^14,15^ and recent reports^16^, serum sEV isolation by precipitation presents the advantages of high recovery and is suitable for small RNA-Seq. Therefore, we used a commercially available precipitation kit for exosome isolation in the present study. The kit efficiently isolated serum sEVs with little contamination from membranous organelles or serum proteins.

### Human, rat, and mouse serum sEVs possessed distinct sizes and particle numbers as well as small RNA contents and might transmit information through differential small RNA patterns

Through nanoparticle tracking analysis, we found that the size and number of serum sEVs varied between the species. The size of human sEVs was the largest, but the number of sEVs per unit volume of serum and the content of sEV small RNA were lowest in humans. The size of mouse sEVs was the smallest, but the number of sEVs per unit volume of serum and the content of sEV small RNA were highest in mice. These characteristics of rat serum sEVs were intermediate (Fig. 1B, Fig. 2).

Through small RNA sequencing, we found that serum sEVs contained not only abundant miRNA but also a considerable amount of tRNA fragments (tRFs & tiRNAs). Further analyses revealed that although human serum presented the lowest number of sEVs and the lowest total amount of sEV small RNA, the ratio of miRNAs in small RNAs was highest in humans (reaching 52.1%). Mouse serum exhibited the greatest number of sEVs and the highest total content of small RNA, but the ratio of miRNAs was lowest in mice (only 12.9%), and the other annotated small RNAs mostly consisted of tRNA fragments (Fig. 3). Compared to human sEVs, rat and mouse serum sEVs presented a much higher content of tRNA fragments (tRFs & tiRNAs) than miRNAs, which suggests that rat and mouse serum sEVs might transmit information through differential small RNA patterns.

On the other hand, we propose that the significant increase in tRNA fragments in mouse and rat serum sEVs might correspond to the shorter life cycle and more vigorous metabolism of mice and rats^17^, leading somatic cells to generate and discard more intracellular RNA fragments via sEVs. As early hypotheses and recent findings have suggested, sEVs might also function as cellular garbage bags that expel unusable or even harmful cellular constituents from cells^18,19^.

### Most serum miRNAs existed both inside and outside of the sEVs but were enriched in sEVs

The miRNA proportions both inside and outside of sEVs were highest in humans among the examined species (Fig. 5A). Although the types of serum sEV miRNAs overlapped with those outside of serum sEVs, most miRNAs were enriched inside serum sEVs, especially in rats and mice (Fig. 6B). We also identified a few serum sEV-specific miRNAs in humans and mice that were detectable exclusively in serum sEVs and presented a TPM ≥ 10. None of the highly abundant serum miRNAs appeared exclusively outside of sEVs in all three species.

To compare the miRNA data from serum sEVs and those from whole serum in previous studies, the present miRNA sequencing data of serum sEVs were compared to a widely referenced healthy human serum miRNA profiling study^1^. Among the serum miRNAs reported by Chen *et al*., 89.9% (80 out of 89 miRNAs) were detected in the present study (see Supplementary Fig. S3A, and Supplementary Table S8 online). Most of Chen’s serum miRNAs (78 out of 89 miRNAs) were found in serum sEVs, and 62.8% (49 out of 78 miRNAs) of which were enriched in serum sEVs (fold change > 2.0). The abundance of the miRNAs detected in serum sEVs was positively correlated with that in Chen’s study (see Supplementary Fig. S3B online).

These observations affirmed the reproducibility of the detection of serum miRNAs among different populations by independent research groups, verified the enrichment of miRNAs in serum sEVs, and supported the possibility and necessity of using serum sEV miRNAs as biomarkers with increased sensitivity^4,20^. However, the present study only compared the components of miRNAs between serum sEVs and sEV-depleted serum from healthy individuals. It is important to expand this work to examine the differences under pathological conditions.

### Common and unique serum sEV miRNAs among humans, rats and mice

For translational studies, it is essential to determine the common serum sEV miRNAs among humans, rats, and mice. In the present study, a total of 188 serum sEV miRNAs that existed in all three species were identified as common miRNAs (see Supplementary Table S6 online). These common serum miRNAs were generally high in abundance. However, the relative expression of these common miRNAs was still different between the species (Fig. 6D). Consequently, when using a rat or mouse model to study the biological significance or biofunctions of serum sEV miRNAs, we would recommend focusing on serum sEV miRNAs of higher abundance that are shared among species.

### Serum sEVs may contain miRNAs from tissues and organs throughout the body, with blood cells as the main contributor

By searching the relevant databases and performing a literature review, we found that serum sEV miRNAs may originate from tissues and organs throughout the body, including blood cells, skin, adipose tissue, and various internal organs (Table 4). Hence, the type and quantity of serum sEV miRNAs could reflect the physiological and pathological states of tissues and organs^6,21^.

Although 500, 319 and 446 known miRNAs were detected in human, rat and mouse serum sEVs, respectively, the top 10 serum sEV miRNAs accounted for 93.0%, 84.2% and 67.3% of all detectable miRNAs in humans, rats and mice, respectively. Compared to humans and rats, the category of miRNAs carried by serum sEVs showed more diversity in mice. Even more impressively, the most abundant miRNAs in serum sEVs came from blood cells. In human serum, miR-486-5p and miR-92a-3p accounted for 69.6% and 8.7% of the total serum sEV miRNAs, respectively (Fig. 7). It is reasonable for blood cells to be the main cellular components of blood, and the sEVs released by blood cells carrying certain miRNAs were found to be important contributors to circulating cell-free miRNAs. Therefore, in the assessment of serum sEV miRNAs as diagnostic or prognostic markers, we should not neglect the contribution of blood cells.

A number of published serum diagnostic markers are blood cell-enriched miRNAs (e.g., miR-486-5p and miR-92a-3p). PubMed searches revealed more than 40 manuscripts published between September 2013 and April 2019 that reported either miR-486-5p or miR-92a-3p as a serum biomarker for cancer, inflammatory conditions or immune disorders^22–27^. Based on the present study, we propose that efforts to convert these miRNAs into diagnostic markers or therapeutic targets should be approached with great caution, as they may reflect a blood cell-based phenomenon rather than a pathological condition. However, because of their rare characteristic of travelling throughout the body, blood cells could act as very important messengers or regulators in either physiological or pathological conditions. The biofunctions of these blood cell-originating top serum sEV miRNAs deserve further investigation.

### The potential role of serum sEV tRFs & tiRNAs

In the present study, we found considerable tRNA contents (tRFs & tiRNAs) in serum sEVs from all three species. tiRNAs are produced by specific cleavage in the anticodon loop of mature tRNA to generate 5′-tRNA and 3′-tRNA halves (30-35 nt), and tRFs are fragments derived from tRNA or pre-tRNA (15–30 nt)^28,29^. These tRNAs are known to act as microRNAs in RNA interference^29^ and to inhibit protein synthesis^30^. It has been reported that the composition and abundance of tRFs and tiRNAs vary with disease conditions such as those associated with cancers^31,32^, acquired metabolic disorders^33^, neurological disorders^34^, and pathogen infections^35^. However, tRNA (tRFs & tiRNAs) populations are highly enriched in biofluids, sometimes to levels higher than those of microRNAs^36^. Although miRNAs are the main candidate for biofluid-based biomarker discovery at present, considering the high abundance of tRFs and tiRNAs in body fluids^36^, their involvement in pathological processes^31,32^, and the ability to discriminate cancer patients from healthy controls according to their analysis^37^, it is possible to develop tRF- and tiRNA-based noninvasive biomarkers. Thus, the roles of tRNA components in serum sEVs and their application value in translational medicine merit close attention.

In conclusion, our findings not only confirmed the rationality of considering serum sEV miRNAs as potential noninvasive diagnostic markers but also suggested the research potential of serum sEV tRNAs (tRFs & tiRNAs). Most importantly, the present study revealed great differences in the composition of serum sEV small RNAs between humans, rats, and mice and suggested that mice and rats might exhibit differentially biased mechanisms for physiological and pathological regulation. Inadequate attention to these differences could hinder the clinical translation of animal model-based studies. To maximize the relevance of our basic research to the clinical setting, more human-based research should be integrated into current serum work addressing sEV miRNAs as biomarkers and therapeutic targets.

## Materials and Methods

### Volunteer recruitment

The human blood used in this investigation was obtained from nine healthy volunteers (5 were female, and 4 were male). The median age of the volunteers was 29 years and ranged from 21 years to 43 years. The blood collection and preparation protocols were approved by the Ethics Committee of the Affiliated Hospital of Nantong University. Written informed consent was obtained from each volunteer. All methods were performed in accordance with the relevant guidelines and regulations.

### Animal experiments

Male C57BL/6 mice and male SD rats were housed in the animal facility of Nantong University. All animal experimental protocols were approved by the Animal Ethics Committee of Nantong University. Animal care and experiments were performed in line with the relevant guidelines and regulations.

### Serum preparation

Blood was collected from healthy volunteers by standard antecubital vein phlebotomy using serum vacuum tubes (BD Vacutainer, 367814). The collected whole blood was left undisturbed for 1 h at room temperature and 2 h at 4 °C and then centrifuged at 1,000 g at 4 °C for 10 min. The resulting serum was transferred to a clean tube and centrifuged again at 2,000 g before aliquoting and storage at −80 °C.

Blood was collected by left ventricular or orbital puncture from male SD rats (200–250 g, 8 weeks) and C57BL/6 mice (20–25 g, 8 weeks). To prevent hemolysis, aspiration was performed slowly and evenly. Serum was obtained via the same protocol used for human serum preparation.

### Isolation of small extracellular vesicles (sEVs, exosomes)

Serum samples were thawed on ice. Pools of 4 to 5 samples (equal-volume) from the same species were used for these experiments. The starting volume of the pooled serum for all sEV isolation experiments was 500 µl. Each 500 µl pooled serum sample was centrifuged at 21,000 g at 4 °C for 15 min to remove debris and large EVs^38^. Exosome-enriched sEV fractions were precipitated by using ExoQuick (System Biosciences Inc., Mountain View, CA) according to the manufacturer’s instructions. Briefly, 1/4 volume of ExoQuick solution was added to the sera, and the samples were incubated at 4 °C for 40 min. The mixture was then centrifuged at 1,500 g for 30 min. The pelleted sEV fraction was resuspended in 100 μl particle-free PBS (Sigma, P4417). The supernatant was collected as sEV-depleted serum. In some cases, serum sEVs, sEV-depleted serum, or whole serum was lysed in RIPA (Beyotime Biotechnology, Shanghai, China) for protein sample preparation or TRIzol (Life Technologies, Carlsbad, CA) for RNA sample preparation.

### Nanoparticle tracking analysis (NTA)

The size and particle concentration of the serum sEVs and particles in sEV-depleted serum were measured by NTA (NanoSight NS300, Malvern, UK) as described previously^14^. Samples were diluted 2,000-fold in particle-free PBS. The measurement time was 60 s, and the number of frames per second was 25. Triplicate measurements were obtained from each sample.

### Transmission electron microscopy

Serum sEVs were visualized using transmission electron microscopy (TEM)^39^. Briefly, 30 μl of an sEV suspension was mixed with 30 μl of 4% paraformaldehyde for fixation; 10 μl of this mixture was transferred to each of formvar/carbon-coated electron microscopy grids, followed by incubation for 20 min and washing 3 times in PBS. The grids were transferred to a 50 μl drop of 1% glutaraldehyde and fixed for 5 min, then transferred to a 100 μl drop of distilled water and washed for 2 min. To contrast the samples, grids were negatively stained in a 50 μl drop of uranyl-oxalate solution, pH 7.0, for 5 min. Finally, the grid was embedded in a 50 μl drop of methyl-cellulose-UA (a mixture of 4% uranyl acetate and 2% methylcellulose in a ratio of 100 ml/900 ml, respectively) for 10 min on ice in the dark and air-dried. The preparations were examined by TEM (HT7700, Hitachi Ltd., Tokyo, Japan) at an accelerating voltage of 80.0 kV.

### Western blotting

sEV samples were lysed in ice-cold RIPA buffer (Beyotime, China) on ice for 15 min and centrifuged at 13,000 g for 10 min. The protein concentration in the supernatant was determined via the BCA assay (Pierce, NCI225CH). Thirty micrograms of total protein was separated by 12% sodium dodecyl sulfate-polyacrylamide gel electrophoresis and electroblotted onto a PVDF membrane (Millipore, USA). The membrane was blocked with 5% nonfat milk PBST for 1 h at RT and then incubated with primary antibodies against CD63 (rabbit polyclonal, System Biosciences, EXOAB-CD63A-1), CD81 (rabbit recombinant monoclonal, Abcam, ab109201), CD9 (rabbit polyclonal, System Biosciences, EXOAB-CD9A-1), calnexin (rabbit polyclonal, Proteintech, 10427-2-Ap) and albumin (rabbit polyclonal, Proteintech, 16475-1-AP) overnight. After incubation with a goat anti-rabbit HRP secondary antibody (Jackson Immunoresearch, West Grove, PA, 111-035-003) for 1 h at RT, protein bands were visualized using an enhanced chemiluminescent (ECL) substrate (Tanon, Shanghai, China, 180–501).

### RNA extraction and characterization

Total RNA was extracted from serum sEVs and sEV-depleted serum samples using TRIzol reagent (Life Technologies, Carlsbad, CA) according to the manufacturer’s instructions. During RNA sample preparation, 50 fmol of synthetic *Caenorhabditis elegans* (cel)-miR-39-3p was spiked in and used to normalize the technical variation between the samples. The RNA pellet was resuspended in 30 µl of RNase-free water and analyzed with a Nanodrop spectrophotometer (Thermo Fisher Scientific) and an Agilent Bioanalyzer 2100 system (Agilent, Palo Alto, CA).

### Small RNA library construction and deep sequencing

Serum sEV RNA and sEV-depleted serum RNA were profiled by small RNA sequencing conducted by OE Biotech Co., Ltd. (Shanghai, China) with biological replicates for each group. Small RNA libraries were generated using an Illumina TruSeq Small RNA Sample kit (Illumina, San Diego, CA, RS-200-0012). As per the manufacturer’s recommendations, total RNA was subjected to sequential 3ʹ and 5ʹ adapter ligations (T4 RNA Ligase 2, Epicenter, LR2D1132K) followed by reverse transcription (SuperScript II Reverse Transcriptase, Invitrogen, 18064–014) into a cDNA library. The amplified cDNAs were purified using 6% Novex TBE PAGE gels (Invitrogen, EC6265BOX), and bands between 147 nt and 157 nt that contained RNA fragments of 22 nt to 30 nt (corresponding to miRNA) were cut out. Library quality was assessed in an Agilent Bioanalyzer 2100 system (Agilent). The qualified cDNA libraries were used for cluster generation and sequenced on the Illumina HiSeq. 2500 platform (Illumina) to obtain 125 bp single-end reads.

### Sequencing data analysis and validation

Small RNA sequencing data analyses were performed as described in Supplementary Fig. S4. First, by using Cutadapt (version 1.7.1) and the FASTX Toolkit (version 0.0.13), adaptors as well as low-quality reads (Q30 < 80%) and low-complexity reads were removed from the raw reads (the total unfiltered reads obtained from the HiSeq. 2500 platform), and the remaining reads were designated as clean reads. Thereafter, reads shorter than 15 nt or longer than 41 nt were filtered from these clean reads to obtain valid reads. To annotate the known miRNAs, these filtered reads were subjected to Blast searches against miRNA sequences downloaded from miRBase (Release 22.0). The unmapped reads were aligned against Rfam (version 11.0) using Blastn software to annotate the tRNA, rRNA, snRNA, and Cis-reg species. The reads filtered by Rfam were aligned against the corresponding genome and repeat databases to identify degraded mRNA fragments (gene) and repeats. We also tried to annotate known tRFs and tiRNAs. The valid reads were subjected to Blast searches against tRF and tiRNA sequences downloaded from GtRNAdb (Genomic tRNA Database Release 18.1) and tRFdb (A relational database of Transfer RNA related Fragments)^40^. The length distribution and unique reads were generated from the obtained valid reads and mapped reads. The expression of miRNAs was normalized as transcripts per million reads (TPM). Trimmed sequencing reads were deposited in the European Nucleotide Archive (data for humans, https://www.ebi.ac.uk/arrayexpress/experiments/E-MTAB-8376; data for rats and mice, https://www.ebi.ac.uk/arrayexpress/experiments/E-MTAB-8377).

The expression of miRNAs in serum sEVs and sEV-depleted serum was validated by quantitative reverse transcription polymerase chain reaction (qRT-PCR) as described previously^13^ using the same batch of samples prepared for small RNA sequencing. Six miRNAs (miR-125a-5p, miR-125b-5p, miR-191-5p, miR-27b-3p, miR-486-5p and miR-99a-5p) with different levels of expression were selected for validation. The primers used for RT-PCR are provided in Supplementary Table S9.

### Statistical analysis

The data are presented as the mean ± SD. Comparisons between means were assessed by Student’s t-test or one-way ANOVA using GraphPad Prism (Prism 5.0; https://www.graphpad.com/scientific-software/prism/). P values of less than 0.05 were regarded as statistically significant. In some cases, the miRNA expression in serum sEVs and sEV-depleted serum was compared, and differentially expressed miRNAs were identified on the basis of a fold change ≥2 or ≤0.5. Except for small RNA sequencing, which included two biological replicates for each group, the data provided in the present study came from three or more independent experiments.

## Supplementary information

Supplementary information.

Supplementary information2.

Supplementary information3.

Supplementary information4.

Supplementary information5.

Supplementary information6.

Supplementary information7.

Supplementary information8.

Supplementary information9.

Supplementary information10.

## References

[1] Chen X (2008). Characterization of microRNAs in serum: a novel class of biomarkers for diagnosis of cancer and other diseases. Cell research.

[2] Johnstone RM, Adam M, Hammond JR, Orr L, Turbide C (1987). Vesicle formation during reticulocyte maturation. Association of plasma membrane activities with released vesicles (exosomes). The Journal of biological chemistry.

[3] Valadi H (2007). Exosome-mediated transfer of mRNAs and microRNAs is a novel mechanism of genetic exchange between cells. Nature cell biology.

[4] Cheng Lesley, Sharples Robyn A., Scicluna Benjamin J., Hill Andrew F. (2014). Exosomes provide a protective and enriched source of miRNA for biomarker profiling compared to intracellular and cell-free blood. Journal of Extracellular Vesicles.

[5] Chen F (2015). Effects of Focal Cerebral Ischemia on Exosomal Versus Serum miR126. Translational stroke research.

[6] Bala S (2012). Circulating microRNAs in exosomes indicate hepatocyte injury and inflammation in alcoholic, drug-induced, and inflammatory liver diseases. Hepatology.

[7] Saha B (2018). Extracellular vesicles from mice with alcoholic liver disease carry a distinct protein cargo and induce macrophage activation through heat shock protein 90. Hepatology.

[8] Mitchell PS (2008). Circulating microRNAs as stable blood-based markers for cancer detection. Proceedings of the National Academy of Sciences of the United States of America.

[9] Thery C (2018). Minimal information for studies of extracellular vesicles 2018 (MISEV2018): a position statement of the International Society for Extracellular Vesicles and update of the MISEV2014 guidelines. Journal of extracellular vesicles.

[10] Vickers KC, Palmisano BT, Shoucri BM, Shamburek RD, Remaley AT (2011). MicroRNAs are transported in plasma and delivered to recipient cells by high-density lipoproteins. Nature cell biology.

[11] Arroyo JD (2011). Argonaute2 complexes carry a population of circulating microRNAs independent of vesicles in human plasma. Proceedings of the National Academy of Sciences of the United States of America.

[12] Kuksa PP (2019). DASHR 2.0: integrated database of human small non-coding RNA genes and mature products. Bioinformatics.

[13] Minami K (2014). miRNA expression atlas in male rat. Scientific data.

[14] Ji Q (2016). Increased Brain-Specific MiR-9 and MiR-124 in the Serum Exosomes of Acute Ischemic Stroke Patients. PloS one.

[15] Zhou X (2017). Characterization of mouse serum exosomal small RNA content: The origins and their roles in modulating inflammatory response. Oncotarget.

[16] Buschmann D (2018). Evaluation of serum extracellular vesicle isolation methods for profiling miRNAs by next-generation sequencing. Journal of extracellular vesicles.

[17] Speakman J. R. (2005). Body size, energy metabolism and lifespan. Journal of Experimental Biology.

[18] Raposo G, Stoorvogel W (2013). Extracellular vesicles: exosomes, microvesicles, and friends. The Journal of cell biology.

[19] Takahashi A (2017). Exosomes maintain cellular homeostasis by excreting harmful DNA from cells. Nature communications.

[20] Szabo G, Momen-Heravi F (2017). Extracellular vesicles in liver disease and potential as biomarkers and therapeutic targets. Nature reviews. Gastroenterology & hepatology.

[21] Vallabhajosyula Prashanth, Korutla Laxminarayana, Habertheuer Andreas, Yu Ming, Rostami Susan, Yuan Chao-Xing, Reddy Sanjana, Liu Chengyang, Korutla Varun, Koeberlein Brigitte, Trofe-Clark Jennifer, Rickels Michael R., Naji Ali (2017). Tissue-specific exosome biomarkers for noninvasively monitoring immunologic rejection of transplanted tissue. Journal of Clinical Investigation.

[22] Tian F (2016). Aberrant miR-181b-5p and miR-486-5p expression in serum and tissue of non-small cell lung cancer. Gene.

[23] Ji X (2015). The Anti-fibrotic Effects and Mechanisms of MicroRNA-486-5p in Pulmonary Fibrosis. Scientific reports.

[24] Regev K (2017). Association Between Serum MicroRNAs and Magnetic Resonance Imaging Measures of Multiple Sclerosis Severity. JAMA neurology.

[25] Cun J, Yang Q (2018). Bioinformatics-based interaction analysis of miR-92a-3p and key genes in tamoxifen-resistant breast cancer cells. Biomedicine & pharmacotherapy = Biomedecine & pharmacotherapie.

[26] Kong R, Gao J, Si Y, Zhao D (2017). Combination of circulating miR-19b-3p, miR-122-5p and miR-486-5p expressions correlates with risk and disease severity of knee osteoarthritis. American journal of translational research.

[27] Li C (2018). Serum miR-486-5p as a diagnostic marker in cervical cancer: with investigation of potential mechanisms. BMC cancer.

[28] Anderson P, Ivanov P (2014). tRNA fragments in human health and disease. FEBS letters.

[29] Garcia-Silva MR, Cabrera-Cabrera F, Guida MC, Cayota A (2012). Hints of tRNA-Derived Small RNAs Role in RNA Silencing Mechanisms. Genes.

[30] Sobala A, Hutvagner G (2013). Small RNAs derived from the 5′ end of tRNA can inhibit protein translation in human cells. RNA biology.

[31] Maute RL (2013). tRNA-derived microRNA modulates proliferation and the DNA damage response and is down-regulated in B cell lymphoma. Proceedings of the National Academy of Sciences of the United States of America.

[32] Goodarzi H (2015). Endogenous tRNA-Derived Fragments Suppress Breast Cancer Progression via YBX1 Displacement. Cell.

[33] Chen Q (2016). Sperm tsRNAs contribute to intergenerational inheritance of an acquired metabolic disorder. Science.

[34] Hanada T (2013). CLP1 links tRNA metabolism to progressive motor-neuron loss. Nature.

[35] Selitsky SR (2015). Small tRNA-derived RNAs are increased and more abundant than microRNAs in chronic hepatitis B and C. Scientific reports.

[36] Dhahbi JM (2013). 5′ tRNA halves are present as abundant complexes in serum, concentrated in blood cells, and modulated by aging and calorie restriction. BMC genomics.

[37] Telonis AG (2015). Dissecting tRNA-derived fragment complexities using personalized transcriptomes reveals novel fragment classes and unexpected dependencies. Oncotarget.

[38] Witwer Kenneth W., Buzás Edit I., Bemis Lynne T., Bora Adriana, Lässer Cecilia, Lötvall Jan, Nolte-‘t Hoen Esther N., Piper Melissa G., Sivaraman Sarada, Skog Johan, Théry Clotilde, Wauben Marca H., Hochberg Fred (2013). Standardization of sample collection, isolation and analysis methods in extracellular vesicle research. Journal of Extracellular Vesicles.

[39] Thery C, Amigorena S, Raposo G, Clayton A (2006). Isolation and characterization of exosomes from cell culture supernatants and biological fluids. Current protocols in cell biology / editorial board, Juan S. Bonifacino… [et al.] Chapter 3, Unit.

[40] Kumar P, Mudunuri SB, Anaya J, Dutta A (2015). tRFdb: a database for transfer RNA fragments. Nucleic acids research.

