# Supplementary material for: Characterization of serum small extracellular vesicles and their small RNA contents across humans, rats, and mice
Source: Sci Rep. 2020 Mar 6;10:4197. doi: 10.1038/s41598-020-61098-9 (PMC7060188; doi:10.1038/s41598-020-61098-9)
Supplement: Supplementary file 1 — Supplementary information. [file 41598_2020_61098_MOESM1_ESM.docx]

**Supplementary Table 1 The concentration and size distribution of serum sEV from human, rat and mouse**

|  | **Serum sEV** | | **De-sEV serum** | | **Serum** | |
| --- | --- | --- | --- | --- | --- | --- |
|  | Concentration  (particles/ml) | Diameters (nm) | Concentration  (particles/ml) | Diameters (nm) | Concentration  (particles/ml) | Diameters (nm) |
| Human | 1.12 ± 0.09 ×10^12^ | 112.7 ± 30.3 | 8.96 ± 0.218 × 10^9^ | 146.6 ± 54.2 | 2.72 ± 0.11 × 10^12^ | 120.6 ± 55.0 |
| Rat | 1.29 ± 0.03 × 10^12^ | 99.2 ± 34.0 | 2.03 ± 0.87 × 10^10^ | 98.2 ± 38.9 | 1.44 ± 0.26 × 10^12^ | 130.0 ± 38.1 |
| Mouse | 2.50 ± 0.82 × 10^12^ | 72.7 ± 28.4 | 8.84 ± 1.75 ×10^9^ | 154.8 ± 58.0 | 3.12 ± 0.10 × 10^12^ | 105.3 ± 34.6 |

Note: sEV, small extracellular vesicle; de-sEV, small extracellular vesicle depleted.
