# Supplementary material for: Characterization of serum small extracellular vesicles and their small RNA contents across humans, rats, and mice
Source: Sci Rep. 2020 Mar 6;10:4197. doi: 10.1038/s41598-020-61098-9 (PMC7060188; doi:10.1038/s41598-020-61098-9)
Supplement: Supplementary file 8 — Supplementary information8. [file 41598_2020_61098_MOESM8_ESM.docx]

**Supplementary Table 8 Comparison of human serum sEV miRNAs and de-sEV serum miRNAs to the normal serum miRNAs reported by Chen *et al***

| **miRNA_id** | **Copy number in FS *** | **Copy number in MS *** | **he_TPM** | **hd_TPM** | **Fold-change** |
| --- | --- | --- | --- | --- | --- |
| hsa-miR-486-5p | 3402 | 5389 | 360870.0 | 325558.5 | 1.1 |
| hsa-miR-92a-3p | 370 | 309 | 44958.0 | 10094.5 | 4.5 |
| hsa-miR-451a | 66604 | 49995 | 24460.0 | 3169.0 | 7.7 |
| hsa-miR-423-5p | 193 | 203 | 23392.0 | 28184.0 | 0.8 |
| hsa-miR-191-5p | 601 | 673 | 8452.0 | 3218.0 | 2.6 |
| hsa-miR-26a-5p | 414 | 687 | 5138.0 | 633.0 | 8.1 |
| hsa-miR-146a-5p | 103 | 114 | 2269.5 | 425.5 | 5.3 |
| hsa-miR-22-3p | 170 | 166 | 1927.0 | 610.0 | 3.2 |
| hsa-miR-25-3p | 735 | 742 | 1686.5 | 560.0 | 3.0 |
| hsa-let-7a-5p | 557 | 3373 | 1178.0 | 188.0 | 6.3 |
| hsa-miR-484 | 20 | 51 | 1148.0 | 794.0 | 1.4 |
| hsa-miR-363-3p | 259 | 363 | 1128.5 | 282.0 | 4.0 |
| hsa-miR-320a | 408 | 645 | 1029.5 | 451.0 | 2.3 |
| hsa-let-7f-5p | 1518 | 2500 | 970.5 | 153.5 | 6.3 |
| hsa-miR-151a-5p | 141 | 259 | 962.5 | 222.5 | 4.3 |
| hsa-let-7d-3p | 110 | 188 | 738.0 | 91.0 | 8.1 |
| hsa-miR-96-5p | 33 | 15 | 608.5 | 121.5 | 5.0 |
| hsa-miR-148a-3p | 72 | 95 | 527.0 | 215.0 | 2.5 |
| hsa-miR-186-5p | 276 | 400 | 482.0 | 150.0 | 3.2 |
| hsa-miR-192-5p | 579 | 802 | 455.5 | 192.0 | 2.4 |
| hsa-miR-16-5p | 5532 | 8079 | 446.5 | 41.5 | 10.8 |
| hsa-miR-23a-3p | 11 | 15 | 384.0 | 61.0 | 6.3 |
| hsa-miR-182-5p | 62 | 94 | 359.5 | 29.0 | 12.4 |
| hsa-miR-185-5p | 784 | 2913 | 322.5 | 194.5 | 1.7 |
| hsa-let-7i-5p | 981 | 1169 | 228.5 | 64.5 | 3.5 |
| hsa-let-7g-5p | 1667 | 2450 | 144.5 | 28.5 | 5.1 |
| hsa-miR-151a-3p | 59 | 73 | 124.0 | 108.5 | 1.1 |
| hsa-miR-106b-3p | 1245 | 1557 | 111.5 | 45.0 | 2.5 |
| hsa-miR-24-3p | 29 | 34 | 100.5 | 145.5 | 0.7 |
| hsa-miR-144-3p | 238 | 398 | 92.0 | 23.5 | 3.9 |
| hsa-miR-199a-3p | 11 | 11 | 92.0 | 20.5 | 4.5 |
| hsa-miR-126-3p | 20 | 49 | 71.0 | 20.0 | 3.6 |
| hsa-miR-183-5p | 30 | 44 | 68.0 | 19.0 | 3.6 |
| hsa-miR-148b-3p | 28 | 38 | 64.5 | 6.5 | 9.9 |
| hsa-miR-21-5p | 966 | 901 | 64.5 | 15.0 | 4.3 |
| hsa-miR-500a-3p | 1074 | 931 | 62.0 | 8.5 | 7.3 |
| hsa-miR-146b-5p | 25 | 65 | 58.0 | 8.0 | 7.3 |
| hsa-miR-502-3p | 11 | 12 | 56.0 | 14.5 | 3.9 |
| hsa-miR-30b-5p | 30 | 62 | 47.0 | 17.0 | 2.8 |
| hsa-miR-320b | 40 | 34 | 46.5 | 9.5 | 4.9 |
| hsa-miR-140-3p | 445 | 592 | 44.5 | 16.5 | 2.7 |
| hsa-miR-142-5p | 398 | 700 | 30.5 | 2.5 | 12.2 |
| hsa-let-7b-3p | 643 | 820 | 30.0 | 5.5 | 5.5 |
| hsa-miR-15b-3p | 276 | 362 | 29.0 | 7.5 | 3.9 |
| hsa-miR-223-3p | 32 | 50 | 26.0 | 4.5 | 5.8 |
| hsa-miR-374b-5p | 28 | 21 | 20.5 | 1.0 | 20.5 |
| hsa-miR-378a-3p | 131 | 210 | 16.0 | 39.5 | 0.4 |
| hsa-miR-374a-5p | 32 | 47 | 15.0 | 0.0 | - |
| hsa-miR-424-3p | 20 | 26 | 14.0 | 6.5 | 2.2 |
| hsa-miR-101-3p | 2104 | 2917 | 13.5 | 0.5 | 27.0 |
| hsa-miR-339-5p | 11 | 11 | 13.5 | 7.0 | 1.9 |
| hsa-miR-652-3p | 41 | 50 | 13.5 | 9.0 | 1.5 |
| hsa-miR-576-5p | 22 | 24 | 12.5 | 0.0 | - |
| hsa-miR-107 | 556 | 533 | 10.5 | 8.0 | 1.3 |
| hsa-miR-20a-5p | 1292 | 1676 | 10.5 | 2.0 | 5.3 |
| hsa-miR-26b-3p | 271 | 297 | 10.0 | 1.0 | 10.0 |
| hsa-miR-425-3p | 201 | 264 | 10.0 | 4.0 | 2.5 |
| hsa-miR-18a-3p | 121 | 119 | 9.0 | 3.0 | 3.0 |
| hsa-miR-340-3p | 82 | 136 | 7.5 | 1.5 | 5.0 |
| hsa-miR-194-5p | 50 | 109 | 7.0 | 5.5 | 1.3 |
| hsa-miR-30e-3p | 54 | 104 | 6.5 | 0.0 | - |
| hsa-miR-454-5p | 25 | 19 | 6.5 | 0.0 | - |
| hsa-miR-7-5p | 357 | 596 | 5.5 | 4.0 | 1.4 |
| hsa-miR-532-3p | 21 | 41 | 5.0 | 10.0 | 0.5 |
| hsa-miR-19b-3p | 608 | 359 | 3.5 | 1.5 | 2.3 |
| hsa-miR-130a-3p | 116 | 117 | 2.0 | 0.0 | - |
| hsa-miR-17-5p | 524 | 589 | 2.0 | 0.0 | - |
| hsa-miR-93-3p | 492 | 528 | 2.0 | 0.0 | - |
| hsa-miR-130b-3p | 35 | 83 | 1.5 | 0.0 | - |
| hsa-miR-181a-3p | 31 | 48 | 1.5 | 0.0 | - |
| hsa-miR-29c-3p | 81 | 129 | 1.5 | 0.5 | 3.0 |
| hsa-miR-362-5p | 19 | 33 | 1.5 | 0.0 | - |
| hsa-miR-660-5p | 26 | 31 | 1.5 | 1.5 | 1.0 |
| hsa-miR-106a-3p | 174 | 141 | 0.5 | 0.0 | - |
| hsa-miR-19a-3p | 13 | 24 | 0.5 | 0.0 | - |
| hsa-miR-30c-1-3p | 133 | 215 | 0.5 | 0.0 | - |
| hsa-miR-30d-3p | 150 | 252 | 0.5 | 0.0 | - |
| hsa-miR-98-3p | 50 | 81 | 0.5 | 0.0 | - |
| hsa-miR-210-3p | 17 | 43 | 0.0 | 1.0 | 0.0 |
| hsa-miR-29b-3p | 33 | 16 | 0.0 | 1.0 | 0.0 |
| hsa-miR-142-3p | 222 | 432 | - | - | - |
| hsa-miR-15a | 373 | 557 | - | - | - |
| hsa-miR-18b | 15 | 18 | - | - | - |
| hsa-miR-190 | 14 | 18 | - | - | - |
| hsa-miR-20b | 106 | 91 | - | - | - |
| hsa-miR-32 | 20 | 14 | - | - | - |
| hsa-miR-324-5p | 24 | 17 | - | - | - |
| hsa-miR-590-5p | 15 | 12 | - | - | - |
| hsa-miR-886-5p | 13 | 11 | - | - | - |

Note: * Chen’s 2008 study; FS, female serum; MS, male serum; h, human; sEV, small extracellular vesicle; de-sEV, small extracellular vesicle depleted; e, serum small extracellular vesicle; d, small extracellular vesicle-depleted serum; TPM, transcripts per million reads. The TPM values provided here are the average of two biological replicates.
