# Supplementary material for: Characterization of serum small extracellular vesicles and their small RNA contents across humans, rats, and mice
Source: Sci Rep. 2020 Mar 6;10:4197. doi: 10.1038/s41598-020-61098-9 (PMC7060188; doi:10.1038/s41598-020-61098-9)
Supplement: Supplementary file 9 — Supplementary information9. [file 41598_2020_61098_MOESM9_ESM.docx]

**Supplementary Table 9 Primers for q-RT-PCR**

| miRNA-id | Primer | nt | GC% | Tm ℃ |
| --- | --- | --- | --- | --- |
| miR-99a-5p | AACCCGTAGATCCGATCTTGTG | 22 | 50 | 55 |
| miR-191-5p | CAACGGAATCCCAAAAGCAGCTG | 23 | 52 | 57 |
| miR-125b-5p | TCCCTGAGACCCTAACTTGTGA | 22 | 50 | 55 |
| miR-125a-5p | TCCCTGAGACCCTTTAACCTGTGA | 24 | 50 | 57 |
| miR-486-5p | TCCTGTACTGAGCTGCCCCGA | 21 | 62 | 58 |
| miR-27b-3p | TTCACAGTGGCTAAGTTCTGC | 21 | 48 | 52 |
