# Supplementary material for: Characterization of serum small extracellular vesicles and their small RNA contents across humans, rats, and mice
Source: Sci Rep. 2020 Mar 6;10:4197. doi: 10.1038/s41598-020-61098-9 (PMC7060188; doi:10.1038/s41598-020-61098-9)
Supplement: Supplementary file 10 — Supplementary information10. [file 41598_2020_61098_MOESM10_ESM.docx]

Supplementary Materials

Characterization of serum small extracellular vesicles and their small RNA contents across humans, rats, and mice

Fengbo Zhao ^a, b^, Li Cheng ^a, c^, Qian Shao ^d^, Zixing Chen ^e^, Xiufang Lv ^b, c^, Jing Li ^a^, Li He ^a^, Yufeng Sun ^b, c^, Qiuhong Ji ^f^, Peng Lu ^a, c^, Yuhua Ji ^d, e*^, Juling Ji ^a, c*^

^a^ Department of Pathology, Medical School of Nantong University, Nantong, China; ^b^ Basic Medical Research Center, Medical School of Nantong University, Nantong, China; ^c^ Key Laboratory of Microenvironment and Translational Cancer Research, Nantong, China; ^d^ Key Laboratory of Neuroregeneration of Jiangsu and Ministry of Education, Nantong University, Nantong, China; ^e^ Institute of Immunology, College of Life Science and Technology, Jinan University, Guangdong, China; ^f^ Department of Neurology, Affiliated Hospital of Nantong University, Nantong, China

Fengbo Zhao, Li Cheng, and Qian Shao contributed equally to this work. *Author to whom correspondence should be addressed; E-Mail: [jijuling@ntu.edu.cn](mailto:jijuling@ntu.edu.cn), [tjyh@ntu.edu.cn](mailto:tjyh@ntu.edu.cn)

**Supplementary Figures**


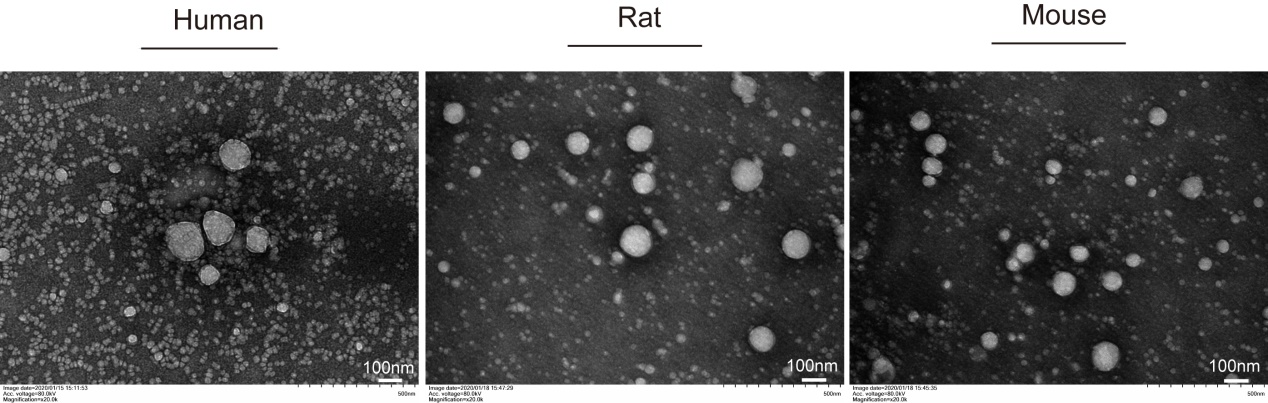


Supplementary Figure 1. Transmission electron microscopy images of sEVs isolated from human, rat and mouse serum by Exoquick precipitation, bar = 100 nm, representative image of sEVs from each species were provided.


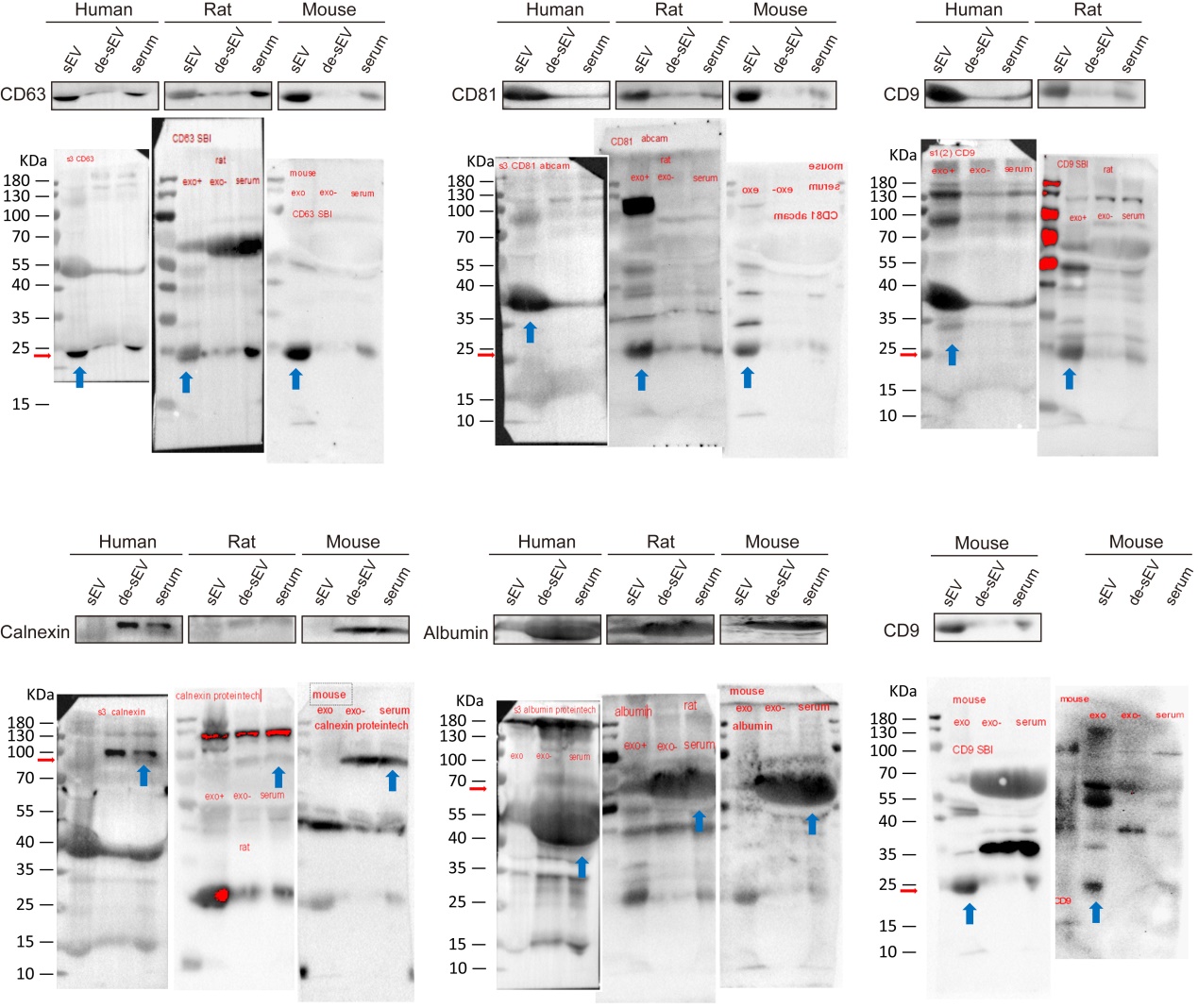


Supplementary Figure 2. The original, uncropped images of Western blot for CD63, CD81, CD9, Calnexin and Albumin. Predicted band size: CD63, 25 KDa; CD81, 26 KDa; CD9, 25 KDa; Calnexin, 90 KDa; Albumin, 69 KDa. An additional blot for mouse CD9 was provided. Red arrow, Predicted band size, blue arrow, observed band. sEV, small extracellular vesicle (exo, exosome); de-sEV, small extracellular vesicle depleted serum (exo-, exosome free serum).


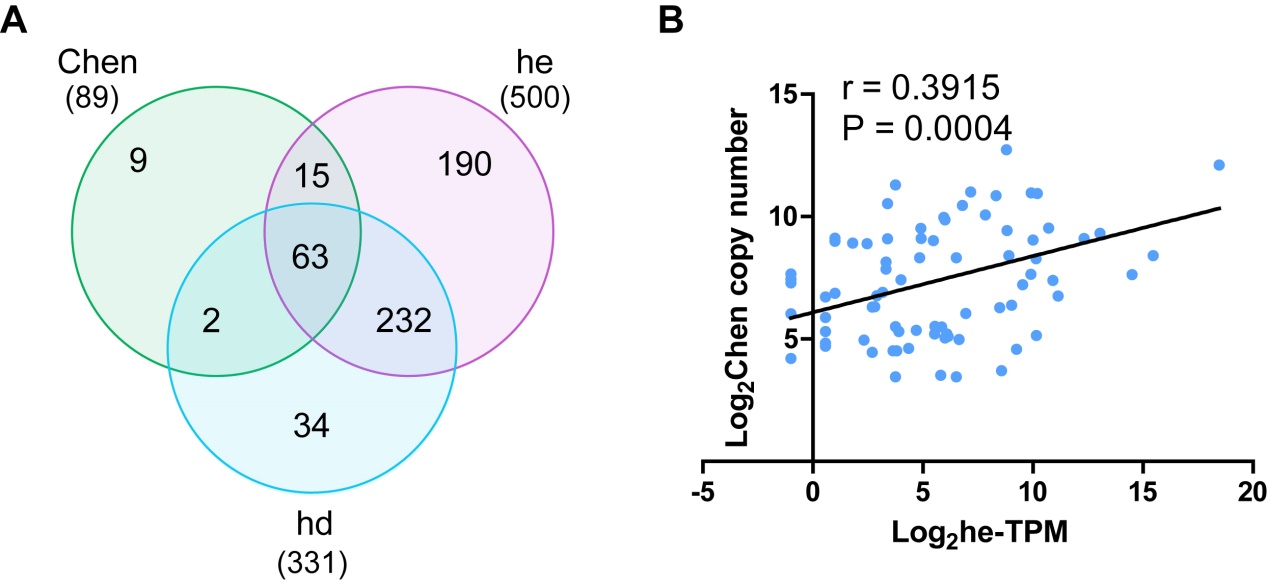


Supplementary Figure 3. Comparison to previously reported human serum miRNAs. Our profiles of serum sEV miRNAs and de-sEV serum miRNAs were compared to a widely recognized study that reported normal human serum miRNA profile by Chen et al. (A) Out of the 89 reported serum miRNAs, 80 were detected in either serum sEV or de-sEV serum, only 2 existed exclusively in de-sEV serum. (B) The abundance of miRNAs detected in serum sEV positively correlated with those in serum reported by Chen’s study. TPM, transcripts per million reads; h, human; e, serum small extracellular vesicle; d, small extracellular vesicle depleted serum.


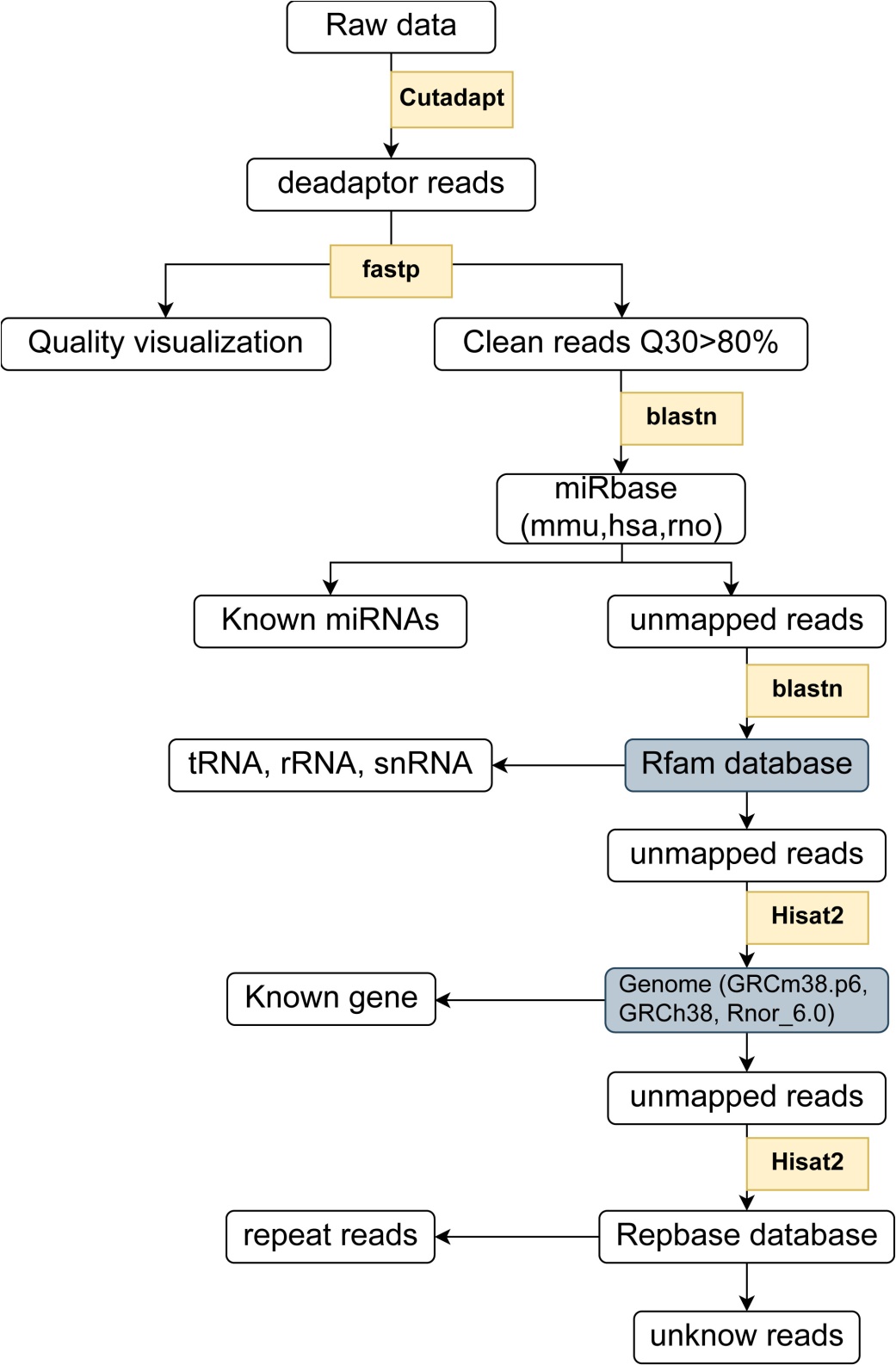


Supplementary Figure 4. Flow chart for small RNAseq data analyses.
